# Predictive robot eyes shape visual attention, performance, and trust in interaction with an industrial CoBot

**DOI:** 10.1038/s41598-026-50476-4

**Published:** 2026-05-04

**Authors:** Lara Naendrup-Poell, Linda Onnasch

**Affiliations:** Technische Universität Berlin, Psychology of Action and Automation, Berlin, 10587 Germany

**Keywords:** Neuroscience, Psychology, Psychology

## Abstract

Collaborative robots increasingly share workspaces with humans, making the predictability of robot actions critical for efficient and safe coordination. Predictive visual cues, such as gaze or arrows, may reduce uncertainty, yet their effectiveness and robustness, especially under failure conditions, remain insufficiently understood. Across two laboratory studies with an industrial CoBot, we examined how predictive cues influence visual attention, prediction, and trust. Participants predicted the movement target of a robotic arm displaying eyes, arrows, or no cues across pre-failure, failure, and post-failure phases, while eye movements, reaction times, and trust were recorded. The second study served as a full replication. Across both studies, gaze cues accelerated visual orienting toward the target, as reflected in earlier target fixations, and improved prediction time when cues were reliable. A cue-action mismatch led to a temporary delayed target fixation and slower responses in both cue conditions. Importantly, performance and attentional guidance largely recovered during subsequent error-free interaction, with gaze cues tending to regain their advantage over no cues. Trust mostly showed the expected dynamic pattern of formation, dissolution, and restoration. Together, the findings demonstrate that predictive cues in industrial HRI enhance action anticipation by guiding visual attention, but their benefits are sensitive to cue reliability.

## Introduction

Industrial work environments have undergone substantial transformation in recent years. Whereas traditional industrial robots were typically enclosed behind safety fences and programmed for repetitive, isolated tasks^1^, contemporary workplaces increasingly rely on collaborative robots (CoBots) that share workspace and interact directly with human workers^2–4^. CoBots are designed to be flexible, adaptable to changing tasks, and capable of safe physical and temporal proximity with human coworkers^1^. This shift aligns with visions of “Industry 5.0,” which emphasize human-centered automation, human-robot teaming, and robotic systems that support rather than replace human capabilities^5–7^. In such settings, robots must not only act safely, but also in ways that are predictable, transparent, and cognitively compatible with human partners. Close-proximity collaboration therefore creates new demands for intuitive coordination and effective communication, which are critical not only for physical safety but also for efficiency and psychological comfort^8^.

Joint action research provides a useful framework for understanding these demands. Joint action is defined as coordinated activity in which two or more agents align their actions in space and time to achieve a shared goal^9^. Successful joint action requires that partners are able to efficiently coordinate their goals, intentions, plans and actions^10^. Michael and Pacherie^11^ identify three key sources of uncertainty that challenge such coordination: motivational uncertainty (whether the partner will engage in the joint action), instrumental uncertainty (how, when, and where the partner will act), and common ground uncertainty (whether shared plans and goals are mutually understood). Communication, whether verbal or non-verbal, plays a central role in reducing these uncertainties by conveying intentions, signaling upcoming actions, and establishing shared expectations^12^. In human-robot interaction (HRI), communication is similarly indispensable as in human interaction^13^. Yet, industrial environments often impose constraints such as noise, time pressure, or limited interaction opportunities, making non-verbal communication particularly valuable.

A fundamental non-verbal communicative mechanism for action prediction in human–human interaction is gaze. Humans automatically interpret others’ gaze direction as an indicator of their attentional focus and likely future actions^14^. Gaze cueing, the rapid shift of one’s attention to the target of another’s gaze, emerges early in development, is highly reflexive, and facilitates efficient prediction and coordination in interactions^14–16^. Importantly, gaze cueing primarily reflects a rapid allocation of visual attention toward a spatial location, which can provide relevant information for subsequent cognitive processes such as action prediction, but is not identical to prediction itself. Research in HRI suggests that robots can evoke similar attentional mechanisms when equipped with gaze-like cues^17,18^, though findings are mixed: Some studies suggest that robot gaze does not always trigger attentional shifts as reliably as human gaze^19^ whereas, other studies have shown that gaze cues can enhance coordination and improve the predictability of robot actions^16,20–22^. Attention can also be guided by non-social, symbolic cues such as arrows^19,23^. From a theoretical perspective, gaze cues are often assumed to rely on an evolutionarily ingrained, biologically relevant mechanism that elicits fast and reflexive orienting responses, whereas arrows, as non-biological, learned symbolic signals^24^, are thought to depend more strongly on voluntary, top-down processing^25^. However, empirical evidence comparing gaze and arrow cueing is mixed. While some studies report differences in cueing strength or underlying attentional processes^24,25^, others suggest largely equivalent effects between biological and non-biological cues. For instance, Gregory and Jackson^26^ found comparable cueing effects for gaze and arrows, with both cue types being modulated by task difficulty and, in some cases, arrows even producing stronger effects. Thus, evidence for a strict dissociation between bottom-up gaze cueing and top-down arrow cueing remains inconclusive.

From a joint action perspective, predictive cues can be conceptualized as information sources that may influence multiple processing stages. Specifically, visual cues can guide attentional allocation (e.g., faster orienting toward task-relevant locations), which in turn can facilitate, but does not guarantee, more efficient action prediction as a higher-level process. In this sense, attentional orienting may serve as a supporting mechanism for prediction and, consequently, for joint action. In industrial HRI, both gaze-like and symbolic cues may therefore function as predictive signals. When implemented effectively, visual predictive cues can orient attention, signal upcoming robot movements, reduce instrumental uncertainty, and facilitate smoother coordination. Thus, their potential benefit for performance is assumed to emerge indirectly via improved access to relevant spatial information. This may be particularly valuable in industrial environments, where fast interpretation of a robot behavior is crucial and auditory communication may be limited.

In addition, conveying information that increases the predictability of a robot’s actions is not only beneficial for immediate task performance but also for shaping trust. Trust in automation is commonly defined as the attitude that an agent will help achieve one’s goals under conditions of uncertainty and vulnerability^27^. According to Hancock et al.^28^, trust is influenced by human-related factors (e.g., attitudes toward robots), robot-related factors (e.g., reliability, predictability, or anthropomorphism), and environmental factors (e.g., task characteristics or communication). Predictive cues may therefore support trust formation indirectly by reducing uncertainty and making robot behavior easier to anticipate. Importantly, trust is dynamic rather than static^29,30^. Typical patterns include trust formation during reliable interaction, trust dissolution following errors, and trust restoration during subsequent error-free phases^30^. These dynamics have been examined in several HRI studies involving robot errors and reliability^31–34^. Although industrial robots have become increasingly reliable, errors still occur in real-world settings^35,36^. For human-centered automation in the sense of Industry 5.0, it is therefore essential to understand not only whether predictive cues support performance and trust during correct operation, but also how robust their effects are when expectations are violated.

Taken together, the present work assumes a sequential relationship in how predictive cues influence behavior: predictive cues provide information, this information can guide visual attention allocation, facilitated attention can support behavioral prediction performance, and repeated success or failure of these predictions can contribute to trust development.

In previous work, we already investigated predictive cues in an industrial HRI scenario^37^. Participants interacted with an industrial robotic arm that moved toward one of several targets while displaying either gaze cues, arrows, or no cues on a screen. Participants had to predict the robot’s upcoming target and select it on a tablet, while eye tracking recorded visual attention. Although predictive cues did not improve prediction time, gaze cues consistently guided participants’ visual attention toward the correct target faster than when no visual cues were present. Arrows, in contrast, did not yield a comparable attentional advantage. Within the present framework, these findings suggest that predictive cues may influence early attentional allocation without necessarily translating into improved prediction performance, indicating that attentional orienting alone may not be sufficient to support higher-level predictive processes. A key limitation of that study was that participants were not explicitly informed that the cues predicted the robot’s actions, and many reported not actively using them. Consequently, the absence of clear benefits for prediction behavior may have been driven by low cue utilization. This, in turn, suggests that the transition from attentional allocation to behavioral prediction may depend on whether cues are noticed, interpreted as informative, and strategically used. Trust results were similarly inconclusive: although cue-movement mismatches occurred during the experiment, many participants failed to notice these errors, again suggesting that the cues were not consistently attended to. From the perspective of the proposed framework, this pattern may indicate that trust is less directly related to attentional processes and may instead depend on whether cue-based predictions are consciously experienced as reliable or violated. If cues are not attended to or utilized, their reliability may not be encoded, and effects on trust may therefore remain limited. Another variable that was assessed in that previous study was workload. Workload was assessed exploratively, as predictive cues could theoretically either reduce workload by facilitating prediction or increase it by adding an additional information source. However, no systematic differences were observed, again potentially due to limited cue usage. Overall, these findings suggested that predictive cues can guide attention, but that their effectiveness critically depends on users noticing, understanding, and employing them.

The present research builds on these findings and addresses the previously identified limitations. To ensure that effects were robust and replicable, we conducted two studies using an improved paradigm, with the second study serving as a full replication. Several key adjustments were implemented to enhance cue clarity, cue usage, and overall task understanding. First, participants were explicitly informed that the gaze cues or arrows indicated the robot’s upcoming movement target. Second, to improve depth interpretation and reduce ambiguity between near and far targets, we modified the cue directions by exaggerating the spatial separation between the coordinates associated with close and distant targets. Whereas the previous study used the robot’s precise three-dimensional target coordinates, which made the depth distinction difficult to perceive according to some participants, the updated cues deliberately amplified these differences to make them more perceptually salient. Third, cue dynamics were simplified: instead of briefly following the robot arm movement first, cues shifted directly toward the intended target and remained fixated, providing a stable and unambiguous predictive signal. Finally, we introduced a demonstration phase in which participants observed the robot executing movements with the corresponding cues (or no cues in the control condition), thereby allowing them to learn the cue-movement contingencies (or only the movement patterns, respectively) before performing the task. Together, these refinements were intended to elicit more consistent cue usage and, in turn, produce more pronounced and reliable effects on attention, prediction, and trust.

Based on previous research, we expected that predictive cues, particularly gaze cues, would support faster target fixation and faster target prediction than having no cues. Importantly, faster target fixation is interpreted as an attentional effect, whereas faster target prediction reflects a higher-level behavioral outcome. We further hypothesized that these benefits would depend on participants’ actual cue usage, such that greater visual attention to the cues would be associated with faster predictions. When cue errors occurred (i.e., mismatches between the cue and the robot’s movement), we expected performance decrements after the cue errors. The misleading predictive signal should cause participants to comply less with the cues in the subsequent phase, thereby slowing down responses relative to a condition without cues and therefore without an error. In terms of accuracy, we did not expect speed-accuracy trade-offs because human gaze and directional cues are highly practiced, can be interpreted rapidly and reliably, and because the task allowed participants to wait for additional kinematic information from the robot if needed. That is, faster responses based on predictive cues should not necessarily come at the cost of reduced accuracy. Therefore, we hypothesized comparable prediction accuracy across conditions. Moreover, faster predictions were expected to improve performance in a secondary, time-constrained memory task that followed each prediction. Regarding trust, we anticipated typical dynamic patterns: trust formation during error-free interaction, trust dissolution after the cue error, and partial trust restoration during subsequent error-free trials, with stronger formation effects in the predictive cue conditions. Finally, workload was assessed again and was not expected to differ systematically across conditions. This expectation reflects competing theoretical mechanisms: predictive cues may reduce workload by facilitating anticipation and reducing uncertainty about upcoming actions, but they may also increase workload by introducing an additional information source that requires processing and integration. Based on this theoretical ambiguity, and prior findings from our previous study that did not reveal systematic workload differences between cue conditions, we did not expect differences in workload across conditions.

## Methods

The following section describes the procedures for Study I and Study II. The second study was a complete replication of Study I, and was conducted to assess the robustness and replicability of the observed effects. Study I was approved by the ethics committee at the Department of Psychology and Ergonomics, Technische Universität Berlin. Because Study II replicated Study I without substantive changes, no additional ethics application was required. All research was conducted in accordance with relevant guidelines and the Declaration of Helsinki. The preregistrations for both studies are available on the Open Science Framework (Study I, Study II). Any deviations from the preregistrations are clearly indicated in the following sections.

### Participants

In Study I, 51 participants were recruited. Nine were excluded (three for not following instructions, five due to missing eye-tracking data caused by technical issues, and one due to over-recruitment), resulting in a final sample size of *N* = 42. This matched the target sample size determined by a G*Power calculation^38^ (*f* =.25, power =.80, $\alpha$ =.05, groups = 3, measurements = 2, correlation =.50, nonsphericity correction = 1). Participants were randomly and evenly assigned to the three conditions, yielding 14 participants per condition. Participants’ ages ranged from 20 to 64 years (*M* = 31.17, *SD* = 9.55). Twenty-five identified as female, 16 as male, and one preferred not to disclose their gender. Most participants were students (27; 15 with a human factors or psychology background), followed by employees (12), two unemployed individuals, and one self-employed participant. In total, 55% reported prior experience with robots, primarily from other experiments and, in some cases, from museums or restaurants. Prior robot experience did not differ between conditions, $\chi ^2(2) = 2.50, p = .29$. Participants received either course credits or monetary compensation. In the eyes and no cue conditions, 9 of 14 participants received monetary compensation, in the arrows condition, 8 of 14 received compensation aligned with German minimum wage, with the remaining participants receiving course credits.

In Study II, the target sample size was again *N* = 42. A total of 52 participants took part, of whom ten were excluded: five due to technical eye-tracking issues, three for not meeting inclusion criteria (one underage, one wearing glasses, one with insufficient German language proficiency), one due to missing the initial trust measurement, and one for not following task instructions. After exclusions, the final sample consisted of 42 participants evenly distributed across the three conditions. Participants’ mean age was 27.45 years (*SD* = 5.73, range: 19–45). Most participants identified as female (29), followed by male (11); two participants did not report their gender. The majority were students (80.9%), with additional participants reporting employment (14%) or unemployment (5%). Of the students, most were enrolled in human factors or psychology programs, with others studying public health, engineering, or business informatics. Approximately 45% reported prior experience with robots. Again, prior experience did not differ between conditions, $\chi ^2(2) = 0.19, p = .91$. Compensation followed the same procedure as in Study I. In each condition, two participants received financial compensation and the remainder received course credit.

### Design

We employed a mixed factorial design with one between-subjects and one within-subjects factor in both studies. The between-subjects factor was predictive cue (abstract anthropomorphic eyes (E), directional arrows (A), no cue (Nc)). The within-subjects factor was interaction experience, consisting of three blocks: block 1 (pre-failure), block 2 (interaction including failure), and block 3 (post-failure).

### Apparatus and task

**Fig. 1:**
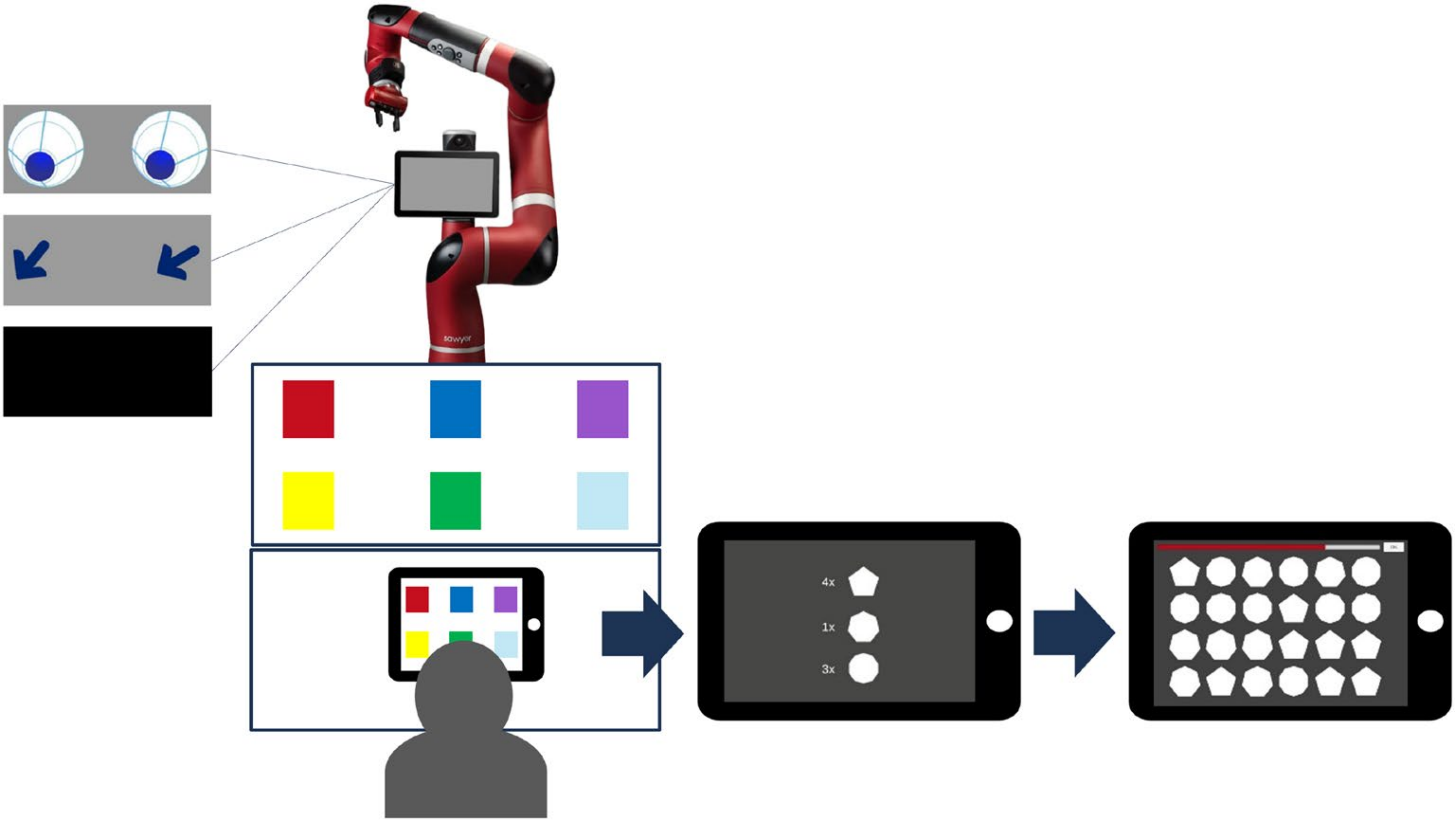
Experimental setting with the robot, showing on the display either eyes, arrows or no cues, the tablet showing the targets and the participant. After selecting the target, participants were shown the number of shapes that had to be selected from the multiple shapes shown on the next screen. At the top of the screen a red bar shows the available time for the memory task.

The experimental setup was adapted from a previous study on predictive cues in human-robot interaction^37^, with slight procedural adjustments. Participants’ primary task was to anticipate the movement target of an industrial CoBot. The robot used was Sawyer (Rethink Robotics), an industrial CoBot designed for collaborative manufacturing contexts, equipped with a seven-degree-of-freedom arm and an integrated display. Mounted on a pedestal, the lower edge of the display was positioned at approximately 135 cm. Depending on condition, the display showed abstract anthropomorphic eyes, arrows, or no visual cues (see Fig. 1). Sawyer and the participant faced each other with two tables positioned between them. On the table in front of the robot, the movement targets were located (six 10 cm $\times$ 10 cm colored squares: red, blue, violet, yellow, green, cyan). In front of the participant, a tablet displayed the same six squares at the beginning of each trial. The tablet was connected via USB-C to the computer controlling the robot, ensuring synchronous operation.

At the start of each trial, participants predicted the robot’s upcoming movement target by selecting the corresponding square on the tablet as quickly and accurately as possible. Prediction speed was functionally relevant, as it directly determined the available time for a subsequent task. After each prediction, participants completed a brief time-constrained memory and visual search task in which they memorized and recalled the number of geometric shapes (pentagon, heptagon, decagon; total count = 8) by selecting the correct quantities from a larger array (see Fig. 1). This secondary task was included to introduce a dual-task demand resembling realistic industrial settings, where operators must coordinate prediction with concurrent cognitive tasks and furthermore to provide participants with an incentive of fast predictions: the faster the predictions, the more time for the memory task. Each trial began with the robot’s arm in an upright, neutral position. In cue conditions, eyes or arrows appeared one second before movement onset and indicated the upcoming target (one of six discrete target locations) throughout the 6 s movement. Unlike in the previous study^37^, the cues did not simulate motion tracking during the reach period but indicated the target statically. During the return phase (5 s), the cues followed the arm’s backward movement until it returned to neutral. The total trial duration was 11 s.

The experiment consisted of three blocks. Blocks 1 and 3 each comprised twelve error-free trials (each square targeted twice; duration: 2 min 12 s). Block 2 consisted of fourteen trials (six correct, two error, six correct; duration: 2 min 34 s). In the predictive cue conditions (eyes and arrows), trials 7 and 8 of block 2 included cue errors in which the visual cue indicated an incorrect target (e.g., cue pointing to cyan while the arm moved to yellow). In the no cue condition, participants completed the same sequence of fourteen trials without misleading visual information. Trial order was randomized once and held constant across participants. A demonstration video is available on the File section of the OSF.

Throughout the experiment, participants wore Pupil Labs Neon eye tracking glasses. The Neon system is a mobile, binocular head-mounted eye tracker that records eye movements with two infrared cameras (200 Hz, 192 $\times$ 192 px) and a scene camera capturing the participant’s field of view (30 Hz, 1600 $\times$ 1200 px, 132° $\times$ 81° FOV)^39^. The glasses were connected to a Motorola Edge 40 Pro running the Neon companion app, which was used for calibration and recording. Data were uploaded to Pupil Cloud, a web-based platform for storage and analysis^39^. To minimize head movements and ensure stable tracking, participants used a chin rest.

### Dependent measures

The primary dependent measures included visual attention allocation, task performance, and trust. All questionnaire-based measures were administered in German.

#### Manipulation check and control variables

As a manipulation check cue usage was assessed via the mean number of fixations within the area of interest (AOI) defined for the robot’s display during block 1. This early phase was chosen to capture initial cue usage before participants could adjust their strategy based on cue reliability.

In addition to the manipulation check, several control measures were assessed to capture individual differences that may shape participants’ perceptions and task behavior. Perceived anthropomorphism in appearance, movement, and context was measured using the Robot Morphology Questionnaire (RoMo-A)^40^. Each of the three subscales (appearance, movement, context) contained three items (e.g., “How human-like is the robot’s external appearance?”) rated from 0% (“not at all human-like”) to 100% (“very human-like”). Internal consistencies were acceptable (appearance: $\omega _{Study1} = .90$, $\omega _{Study2} = .84$; movement: $\omega _{Study1} = .92$, $\omega _{Study2} = .76$; context: $\omega _{Study1} = .88$, $\omega _{Study2} = .84$). The individual tendency to anthropomorphize robots (IDAQ-R) was assessed with a customized four-item version of the Individual Differences in Anthropomorphism Questionnaire based on Waytz et al.^41^. This version, used previously in related work^37,42^, referred specifically to robots (e.g., “To what extent does a robot have intentions?”, “To what extent does a robot experience emotions?”) and was rated on an 11-point Likert scale from 0 (“not at all”) to 10 (“very much”) ($\omega _{Study1} = .81$, $\omega _{Study2} = .81$). To assess mind perception, a four-item scale adapted from Gray and Wegner^43^ was used ($\omega _{Study1} = .89$, $\omega _{Study2} = .87$). Two items assessed the dimension of experience (e.g., “This robot has the capacity to feel pain”), and two assessed agency (e.g., “This robot has the capacity to plan actions”). Ratings were made on a 5-point Likert scale from 1 (“not at all”) to 5 (“extremely”). Participants also completed the Regulatory Focus Scale^44^ ($\omega _{Study1} = .76$, $\omega _{Study2} = .80$), following the 5-point response format proposed by Chang et al.^45^, to capture individual motivational orientations. The scale consists of ten items: five assessing promotion focus (e.g., “I like to do things in a new way”) and five assessing prevention focus (e.g., “I always try to make my work as accurate and error-free as possible”).

After completing the final block, participants were asked whether they had noticed any faulty robot behavior and, if so, to describe it. These responses were used to assess detection of cue errors in block 2. As a subjective measure of cue use, participants also rated how often they relied on the cues (“eyes/arrows”) to predict the robot’s movements on a scale from 1 (“never”) to 4 (“always”). Finally, demographic information was collected, including participants’ age, gender, employment situation, and prior experience with robots.

#### Visual attention allocation

Visual attention allocation was measured as the time to first fixation on the target via eye tracking. This was defined as the time (in ms) from trial onset until the participant fixated the correct target square for the first time in that trial.

#### Performance

Performance was assessed via prediction time (reaction time until correct target selection in ms), prediction accuracy (ratio of correctly selected targets to the total number of selections), and memory task performance. Memory task performance was computed using a composite score based on hit rate and false alarm rate. The hit rate was defined as the number of correctly selected shapes divided by the maximum number of correct shapes (8), and the false alarm rate as the number of incorrectly selected shapes divided by the maximum number of incorrect shapes (16). The resulting performance score was calculated as hit rate minus false alarm rate, yielding a bounded measure ranging from $-1$ (worst possible performance) to $+1$ (best possible performance). This approach was chosen to account for both correct and incorrect selections in contrast to only reporting the number of correctly selected shapes^16^. This composite score penalizes indiscriminate responding (e.g., selecting all shapes) and therefore provides a more conservative and informative measure of performance in this multi-item selection task.

#### Trust

Subjective trust was measured using the “Trust in Automation” subscale of the “Trust in Automation” questionnaire^46^, consisting of two items (“I trust the system”, “I can rely on the system”) rated on a 5-point Likert scale from 1 (“strongly disagree”) to 5 (“strongly agree”). Trust was assessed before the interaction ($t_{0}$) to capture initial trust, after error-free interaction in block 1 ($t_{1}$) to measure trust formation, after block 2 containing the predictive cue error ($t_{2}$) to capture potential trust dissolution, and after block 3 with error-free interaction ($t_{3}$) to assess potential restoration. Pearson correlation between the two items was calculated as a measure of reliability, which ranged from $r_{\text {t3}} = .71$ to $r_{\text {t2}} = .88$ in Study I and $r_{\text {t1}} = .85$ to $r_{\text {t2}} = .89$ in Study II.

#### Workload

Mental workload was assessed after each experimental block using the NASA Task Load Index (NASA-TLX)^47^, which includes six subscales (mental, physical, and temporal demand, performance, effort, frustration) rated on a 21-point scale from very low to very high, corresponding to values from 0 to 100 in increments of 5 ($\omega _{Study1} = .93$, $\omega _{Study2} = .89$). For the analysis, workload was computed as an overall raw (unweighted) score by averaging across all subscales, which is a common scoring approach for the NASA-TLX^48^. In the second study, we additionally implemented a self-developed single-item to examine whether a brief, efficient workload measure could complement the NASA-TLX. The item was: “How stressed did you feel by the overall task in the last experimental block?” and participants responded on a scale from –10 (very underchallenged), through 0 (just right), to +10 (very overchallenged). This item was conceptually guided by the DLR-WAT^49^, a workload instrument specifically developed to assess both underload and overload in complex human-machine systems. The item was administered alongside the NASA-TLX after each experimental block.

#### Exploratory eye tracking measures

In addition to the number of fixations on the robotic display in the first block (manipulation check) and the time to first fixation on the correct target (visual attention allocation measure), further eye-tracking metrics were collected exploratory for certain AOIs. The main focus was on AOIs that provided directional information for participants, namely the robot’s display and the robotic arm. For those, the mean number of fixations and the mean fixation duration (in ms) were assessed. Due to technological constraints, it was not possible to define a dynamic, moving AOI for the robotic arm. Instead, a static AOI covering the entire movement region of the arm was defined. You can find an image of the defined AOIs on the OSF. Fixations were detected using Pupil Labs’ algorithm with a minimum duration threshold of 100 ms^50^.

### Procedure

**Fig. 2:**
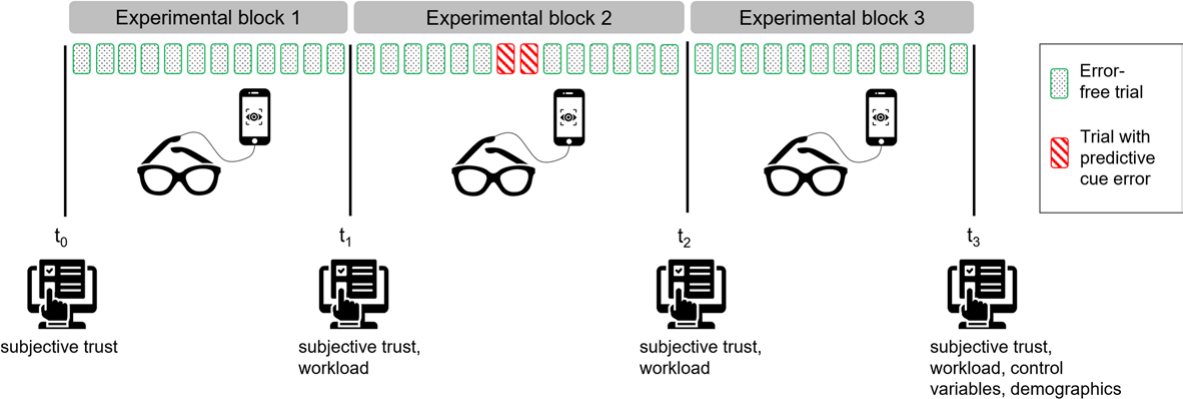
Experimental procedure consisting of three experimental blocks with 12 error free trials each (green-dotted squares) and two additional error trials in block two (red-hatched squares). During the blocks, eye tracking was recorded. Prior to the first block, and after each block subjective measures were assessed.

The studies took place in the department’s laboratory. After providing informed consent, participants were randomly assigned to a condition and received computer-based instructions, including explicit information about the predictive function of the cues. Following initial trust assessment and eye-tracker calibration, participants practiced the memory task and observed a demonstration phase. During this demonstration, the robot sequentially moved its arm toward each target while displaying the corresponding eye or arrow cues (or no cues), depending on the assigned condition. Participants then completed the three experimental blocks. Trust and workload were assessed after each block. Following the final block, participants completed the control measures, and demographic questions, along with the final trust and workload scales. Finally, participants were debriefed about the study’s purpose. The full session lasted approximately 40 minutes. See Fig. 2 for an overview of the experimental procedure.

### Data analysis

Manipulation check and control variables were analyzed using one-way ANOVAs.

Depending on the inferential goal, both frequentist and Bayesian approaches were employed. Analyses corresponding to preregistered hypotheses predicting differences between conditions were conducted using mixed ANOVAs within the null hypothesis significance testing (NHST) framework. For variables for which no differences between conditions were hypothesized, Bayesian ANOVAs were used to quantify evidence in favor of the null hypothesis, as NHST does not allow conclusions about the absence of effects. This analytic choice represents a deviation from the preregistration and was made to better align the statistical approach with the goal of evaluating null effects for the preregistered hypotheses.

Visual attention allocation and task performance were analyzed for each experimental block separately, with block halves reflecting early versus late experience within each interaction phase. This approach allowed examination of adaptation following cue errors in block 2. To maintain analytical consistency, this approach was applied to all blocks. Therefore, three 3 (predictive cue) × 2 (experience: first six vs. last six trials per block) mixed ANOVAs were conducted per experimental block for mean time to first fixation of the correct target, prediction time, and memory task performance. For prediction accuracy, we initially preregistered the same approach with regular ANOVAs, however, considering our hypothesis that conditions would not differ in terms of accuracy, Bayesian ANOVAs were conducted instead to allow quantification of evidence for the null model. This deviation from the preregistration is documented in the updated preregistrations on the OSF. To explore associations between attentional allocation and behavioral performance, Pearson correlations were calculated between prediction time and number of fixations in the AOI Display. Correlations between prediction time and memory task performance were calculated as well.

Trust development across the interaction was analyzed using a 3 (predictive cue) × 4 (measurement time: pre-interaction $t_{0}$, post-block 1 $t_{1}$, post-block 2 $t_{2}$, post-block 3 $t_{3}$) mixed ANOVA, with measurement times mapping onto trust formation, dissolution, and restoration phases. As post hoc analyses, mixed ANOVAs including two measurement time points (e.g., $t_{1}$ vs. $t_{2}$ to further analyze the impact of the cue errors) across the three conditions were conducted.

As we did not expect any differences for workload, a Bayesian ANOVA with a 3 (predictive cue) × 3 (measurement time: post-block 1, post-block 2, post-block 3) mixed design was performed for the NASA-TLX (Study I and II) and the single item (only Study II) to directly assess evidence for the absence of effects. This analytic deviation is likewise documented in the updated preregistrations on the OSF. In addition, correlations of the single item and the NASA-TLX were computed to assess whether both measures are associated and thus potentially capture similar underlying constructs.

All tests were performed using a significance threshold of $\alpha$ =.05, and Bonferroni corrections were applied where multiple comparisons were conducted.

## Results

Because Study II was a direct replication of Study I, results for both studies are reported side by side for each measure to enable immediate comparison of effect patterns and robustness.

### Manipulation check and control variables

As a manipulation check, we examined fixation frequency on the robot’s display during the first experimental block to assess whether participants initially noticed and used the predictive cues (eyes, arrows).

In Study I, participants fixated the display on average 2.12 ($SD = 0.80$) times per trial in the eyes condition, 2.01 ($SD = 0.90$) times in the arrows condition, and 0.58 ($SD = 0.34$) times in the no cue condition. In the replication study, fixation numbers were comparable, with 2.52 ($SD = 1.12$) fixations per trial in the eyes condition, 1.79 ($SD = 1.10$) in the arrows condition, and 0.60 ($SD = 0.27$) in the no cue condition. ANOVAs in both studies showed a significant effect of condition (Study I: $F(2,39) = 19.83$, $p < .001$, $\eta ^2_{p} = .50$; Study II: $F(2,39) = 15.60$, $p < .001$, $\eta ^2_{p} = .44$). Bonferroni corrected post hoc tests demonstrated higher fixation frequencies in both cue conditions compared to the no cue condition (Study I: eyes vs. no cue: $t(39) = -5.65$, $p < .001$; arrows vs. no cue: $t(39) = -5.24$, $p < .001$; Study II: eyes vs. no cue: $t(39) = -5.53$, $p < .001$; arrows vs. no cue: $t(39) = -3.43$, $p = .004$). No significant differences emerged between eyes and arrows (Study I: $t(39) = 0.41$, $p> .999$; Study II: $t(39) = 2.10$, $p = .125$). These results confirm that the predictive cues reliably attracted participants’ attention in both studies.

Descriptive statistics and inferential analyses for all control variables (perceived anthropomorphism in appearance, movement, and context; IDAQ-R; mind perception; and regulatory focus) are presented in Table 1. Only one significant effect emerged: in Study II, perceived anthropomorphism of the context differed between conditions, with the lowest ratings in the no cue condition. No other control variable showed significant group differences. Across both studies, participants generally reported low tendencies to anthropomorphize robots, perceived the robot as rather low anthropomorphic, moderately agentic and low in experience, and reported slightly higher prevention than promotion focus.

**Table 1:** Control variables.

	Eyes *M (SD)*	Arrows *M (SD)*	No cue *M (SD)*	ANOVA
RoMo-A: Appearance	I: 17.83 (20.18) II: 7.69 (5.76)	I: 11.41 (9.31) II: 8.86 (9.28)	I: 9.91 (12.16) II: 9.12 (9.94)	I: $F(2,39) = 1.16$, $p = .324$, $\eta ^2_p = .06$ II: $F(2,39) = 0.11$, $p = .895$, $\eta ^2_p < .01$
RoMo-A: Movement	I: 17.33 (20.35) II: 16.48 (13.25)	I: 21.04 (15.12) II: 20.48 (17.05)	I: 20.00 (20.45) II: 15.00 (9.60)	I: $F(2,39) = 0.15$, $p = .865$, $\eta ^2_p < .01$ II: $F(2,39) = 0.60$, $p = .552$, $\eta ^2_p = .03$
RoMo-A: Context	I: 34.43 (24.09) II: 39.12 (18.01)	I: 35.07 (26.10) II: 39.33 (20.95)	I: 44.74 (26.10) II: 22.21 (15.44)	I: $F(2,39) = 0.84$, $p = .438$, $\eta ^2_p = .04$ II: $F(2,39) = 4.05$, $p = .025$, $\eta ^2_p = .17$
IDAQ-R	I: 1.86 (1.05) II: 1.68 (0.76)	I: 2.14 (1.37) II: 1.45 (0.76)	I: 1.34 (0.56) II: 1.64 (0.69)	I: $F(2,39) = 2.13$, $p = .132$, $\eta ^2_p = .10$ II: $F(2,39) = 0.40$, $p = .673$, $\eta ^2_p = .02$
Mind perception: Agency	I: 2.68 (0.89) II: 2.18 (0.95)	I: 2.68 (1.34) II: 2.79 (1.12)	I: 2.29 (1.16) II: 2.68 (1.05)	I: $F(2,39) = 0.55$, $p = .581$, $\eta ^2_p = .03$ II: $F(2,39) = 1.35$, $p = .271$, $\eta ^2_p = .06$
Mind perception: Experience	I: 1.21 (0.38) II: 1.00 (0.00)	I: 1.11 (0.40) II: 1.00 (0.00)	I: 1.00 (0.00) II: 1.07 (0.27)	I: $F(2,39) = 1.59$, $p = .217$, $\eta ^2_p = .08$ II: $F(2,39) = 1.00$, $p = .377$, $\eta ^2_p = .05$
Regulatory focus: Prevention	I: 4.04 (0.67) II: 4.20 (0.52)	I: 3.79 (0.66) II: 3.96 (0.63)	I: 3.66 (0.62) II: 4.10 (0.59)	I: $F(2,39) = 1.28$, $p = .290$, $\eta ^2_p = .06$ II: $F(2,39) = 0.61$, $p = .547$, $\eta ^2_p = .03$
Regulatory focus: Promotion	I: 3.14 (0.72) II: 3.23 (0.71)	I: 3.10 (0.76) II: 3.20 (0.70)	I: 3.47 (0.56) II: 3.09 (0.79)	I: $F(2,39) = 1.23$, $p = .303$, $\eta ^2_p = .06$ II: $F(2,39) = 0.15$, $p = .863$, $\eta ^2_p < .01$

*Note*. Means (*M*), standard deviations (*SD*) and ANOVA statistics for control variables in Study I and Study II.

#### Cue usage and error detection

In the first study, self-reported cue usage frequency in the eyes condition showed that 5 participants reported always using the cues, 5 used the cues often, 3 sometimes and 1 participant never. In the arrows condition 1 participants used the cues always, 4 often, 6 sometimes and 3 never. In the second study, most participants in the cue conditions also used the cues (13 out of 14 reported “sometimes,” “often,” or “always’), with only one person per condition indicating they never used them.

Regarding error detection in the predictive cue conditions where the cues misguided attention and did not align with the robot’s arm movement, the first study showed that 13 out of 14 participants in the eyes condition and 12 out of 14 in the arrows condition noticed the error. In the replication study, 11 out of 14 participants in both the eyes and arrows conditions correctly identified the mismatch between the cues and the arm movement as faulty robot behavior.

### Visual attention allocation

**Fig. 3:**
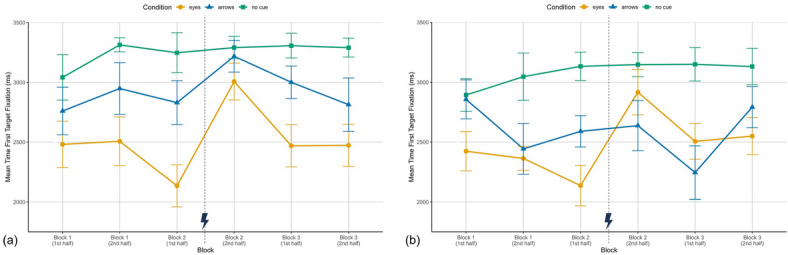
Mean time to first fixation of the correct target in milliseconds in Study I (**a**) and Study II (**b**) over the three experimental blocks, each split in the first six trials and the last six trials, for the three conditions eyes, arrows and no cue. Depicted are means and standard errors. The lightning bolt icon marks the occurrence of cue error trials.

To assess whether predictive cues guide visual attention, we analyzed the mean time from trial onset to the first fixation of the correct target. Time to first target fixation is displayed in Fig. 3 (a) for Study I and (b) for Study II, each showing the three experimental blocks. In Study I, participants in the eyes condition located the correct target fastest ($M_{E} = 2511.98$ ms, $SD = 479.93$ ms), followed by those in the arrows condition ($M_{A} = 2928.82$ ms, $SD = 456.83$ ms), whereas participants without cues were slowest ($M_{Nc} = 3272.63$ ms, $SD = 376.43$ ms). Furthermore, a clear rise of time to first fixation is visible after the occurence of the cue errors in block 2. For the following analyses, one participant in the eyes condition had to be excluded because their gaze data contained fixations only on the targets displayed on the tablet across all blocks (classified as tablet fixations due to insufficient tracking precision), preventing computation of a meaningful target fixation measure. Additionally, one participant in the no cue condition had no valid target fixations in block 1 and was therefore excluded from the block 1 analysis.

In Study II (Fig. 3b), fixation times for the eyes and no cue conditions resembled Study I, while the arrows condition showed a slightly different pattern in block 2: the cue error appeared to affect fixation times only in the eyes condition, not in the arrows condition. Overall means again followed the same ordering as in Study I (Study II: $M_{E} = 2482.22$ ms, $SD = 435.62$ ms; $M_{A} = 2592.18$ ms, $SD = 556.91$ ms; $M_{Nc} = 3042.10$ ms, $SD = 386.42$ ms). Study II also included several missing data points: in block 1 one participant in the arrows condition did not fixate the target before selection; in block 2 two participants in the no cue condition and one in arrows were missing; and in block 3 one participant each from the no cue and arrows conditions were missing due to no registered fixations on the physical targets.

To examine changes within blocks and across conditions, we ran ANOVAs per experimental block on the first six and last six trials. In block 1 both studies showed significant differences between conditions (Study I: $F(2,37) = 4.24$, $p = .022$, $\eta _p^2 = .19$; Study II: $F(2,38) = 4.59$, $p = .016$, $\eta _p^2 = .19$). Bonferroni corrected post hoc tests indicated that participants in the eyes condition were significantly faster than those without cues (Study I: $t(37) = -2.91$, $p = .018$, $d = -.72$; Study II: $t(38) = -3.02$, $p = .013$, $d = -.98$), indicating that eyes accelerated attentional orientation to the target. No other pairwise comparisons were significant (eyes vs. arrows in Study I: $t(37) = -1.56$, $p = .380$, $d = -.51$, Study II: $t(38) = -1.32$, $p = .584$, $d = -.43$; arrows vs. no cue in Study I: $t(37) = -1.40$, $p = .508$, $d = -.46$, Study II: $t(38) = -1.65$, $p = .324$, $d = -.54$). Furthermore, no other effects reached significance in either study (Study I: Experience: $F(1,37) = 2.52$, $p = .121$, $\eta ^2_{p} = .06$, Interaction: $F(2,37) = 0.49$, $p = .616$, $\eta ^2_{p} = .03$; Study II: Experience: $F(1,38) = 0.99$, $p = .326$, $\eta ^2_{p} = .03$, Interaction: $F(2,38) = 2.32$, $p = .112$,$\eta ^2_{p} = .11$).

In block 2, in both studies, the ANOVAs revealed significant main effects of condition (Study I: $F(2,38) = 7.61$, $p = .002$, $\eta _p^2 = .29$; Study II: $F(2,36) = 5.28$, $p = .010$, $\eta _p^2 = .23$) and experience (Study I: $F(1,38) = 19.33$, $p < .001$, $\eta _p^2 = .34$; Study II: $F(1,36) = 9.96$, $p = .003$, $\eta _p^2 = .22$), as well as significant interaction effects (Study I: $F(2,38) = 5.83$, $p = .006$, $\eta _p^2 = .23$; Study II: $F(2,36) = 8.19$, $p = .001$, $\eta _p^2 = .31$). Bonferroni corrected post hoc tests revealed similar overall patterns with some minor differences between the two studies. In the pre-error trials of block 2, participants in the eyes condition were significantly faster than those in the no cue condition in both Study I ($t(38) = -4.46$, $p < .001$, $d = -1.47$) and Study II ($t(36) = -4.89$, $p < .001$, $d = -1.63$). In Study I, participants in the eyes condition were also significantly faster than those in the arrows condition ($t(38) = -2.79$, $p = .025$, $d = -.91$), whereas the arrows and no cue conditions did not differ significantly ($t(38) = -1.71$, $p = .289$, $d = -.56$). In contrast, in Study II, the difference between the eyes and arrows conditions did not reach statistical significance ($t(36) = -2.27$, $p = .088$, $d = -.76$), but participants in the arrows condition were significantly faster than those in the no cue condition ($t(36) = -2.62$, $p = .039$, $d = -.87$). After the cue-error trials (second half of block 2), the differences between conditions disappeared. Participants in the condition with eyes were not significantly faster anymore than participants without cues (Study I: $t(38) = -1.56$, $p = .379$, $d = -.51$; Study II: $t(36) = -0.92$, $p> .999$, $d = -.31$) or with arrows (Study I: $t(38) = -1.16$, $p = .757$, $d = -.38$; Study II: $t(36) = 1.14$, $p = .792$, $d = .38$) and there was no difference between arrows and no cues (Study I: $t(38) = -0.41$, $p> .999$, $d = -.13$; Study II: $t(36) = -1.99$, $p = .161$, $d = -.66$). Furthermore, within-condition comparisons showed that in Study I both predictive cue conditions exhibited significant slowdowns in target fixations after the cue errors (eyes: $t(38) = -4.97$, $p < .001$, $d = -.80$; arrows: $t(38) = -2.30$, $p = .027$, $d = -.37$), while the no cue condition did not change ($t(38) = -0.26$, $p = .800$, $d = -.04$). In Study II, only the eyes condition showed a significantly slower fixation time post-error ($t(36) = -5.27$, $p < .001$, $d = -1.76$), whereas arrows ($t(36) = -0.31$, $p = .760$, $d = -.10$) and no cues ($t(36) = -0.09$, $p = .927$, $d = -.03$) remained unchanged.

In the third experimental block in Study I, the ANOVA revealed a significant effect of condition ($F(2,38) = 8.78$, $p < .001$, $\eta _p^2 = .32$): participants in the eyes condition were significantly faster than those without cues ($t(38) = -4.19$, $p < .001$, $d = -1.36$). The other pairwise comparisons did not reach significance (eyes vs. arrows: $t(38) = -2.21$, $p = .100$, $d = -.72$; arrows vs. no cues: $t(38) = -2.02$, $p = .151$, $d = -.66$). The effects of experience and interaction were also nonsignificant (all $F < 1$). In Study II, condition ($F(2,36) = 5.94$, $p = .006$, $\eta _p^2 = .25$), experience ($F(1,36) = 4.56$, $p = .040$, $\eta _p^2 = .11$), and their interaction ($F(2,36) = 3.84$, $p = .031$, $\eta _p^2 = .18$) were significant. Post hoc tests showed significantly faster fixation times at the beginning of block 3 in both cue conditions relative to no cues (Eyes vs. no cue: $t(36) = -2.71$, $p = .031$, $d = -.90$; arrows vs. no cue: $t(36) = -3.67$, $p = .002$, $d = -1.22$; eyes vs. arrows: $t(36) = 1.08$, $p = .868$, $d = .36$). Across halves of block 3, fixation times increased only for the arrows condition ($t(36) = -3.40$, $p = .002$, $d = -1.13$), while eyes ($t(36) = -0.30$, $p = .770$, $d = -.10$) and no cues ($t(36) = 0.12$, $p = .906$, $d = -.04$) showed no change. In the second half of block 3, the only significant difference was between eyes and no cues ($t(36) = -2.63$, $p = .038$, $d = -.88$), while the other comparisons were not significant (arrows vs. no cue: $t(36) = -1.47$, $p = .448$, $d = -.50$; eyes vs. arrows: $t(36) = -1.07$, $p = .873$, $d = -.36$).

To better assess the magnitude of the observed effects, a mini meta-analytical fixed effects approach following the procedure proposed by Goh et al.^51^ was adopted as an additional exploratory analysis. Effect sizes (*r*) were calculated from the pairwise post hoc comparisons for each study. To obtain a higher-level perspective on cue effects in relation to the occurrence of errors, the data were aggregated into a pre-error phase (comprising block 1 and block 2 pre-error) and a post-error phase (comprising block 2 post-error and block 3). All *r* values were transformed into Fisher’s *z* scores, and weighted mean correlations were computed (see^51^ for details). The resulting aggregated effect sizes are presented in Table 2. In the pre-error phase, the comparison between eyes and no cue yielded a large and significant effect ($r = .53$, $Z = 4.73$, $p < .001$), whereas the arrows versus no cue comparison showed a smaller significant effect ($r = .29$, $Z = 2.58$, $p = .010$). The eyes versus arrows comparison was of similar magnitude ($r = .31$, $Z = 2.76$, $p = .006$), indicating that the advantage of eyes over arrows was comparable to the benefit of arrows over no cues. In the post-error phase, the effect of eyes versus no cue decreased but remained moderate in size ($r = .37$, $Z = 3.25$, $p = .001$). In contrast, the arrows versus no cue effect showed little change in magnitude ($r = .29$, $Z = 2.55$, $p = .011$). The eyes versus arrows comparison decreased to a small non-significant effect ($r = .18$, $Z = 1.62$, $p = .105$). Overall, these results indicate that while both cue types facilitate attention allocation to the target relative to having no cues, the eyes provide a stronger benefit than arrows, and this advantage is more pronounced before the occurrence of an error. In addition, the reduction in effect size after the error is more evident for the eyes condition, suggesting that the impact of the error particularly attenuates the effectiveness of eyes.

**Table 2:** Weighted aggregated time to first target fixation effects pre- and post-error.

	Pre-error			Post-error		
	Eyes–No Cue	Arrows–No Cue	Eyes–Arrows	Eyes–No Cue	Arrows–No Cue	Eyes–Arrows
$r_{Study I}$	0.514	0.246	0.334	0.416	0.192	0.263
$r_{Study II}$	0.540	0.329	0.281	0.308	0.380	0.097
$M_{rz}$	0.586	0.296	0.318	0.383	0.294	0.186
$M_{r}$	0.527	0.288	0.308	0.365	0.286	0.184
Combined *Z*	4.73***	2.58**	2.76**	3.25**	2.55*	1.62

*Note*. $r_{Study I}$ and $r_{Study II}$ denote effect sizes (Pearson’s *r*) derived from the pairwise post hoc comparisons within each study. $M_{rz}$ represents the weighted mean of Fisher’s *z*-transformed correlations, and $M_{r}$ the corresponding back-transformed mean correlation. Combined *Z* values were calculated using Stouffer’s method across the two studies. Pre-error comprises block 1 and block 2 (pre-error), whereas post-error comprises block 2 (post-error) and block 3. Positive values indicate shorter time to first target fixation in the first-mentioned condition of each comparison. *$p < .05$. **$p < .01$. ***$p < .001$.

### Performance

#### Prediction time and accuracy

**Fig. 4:**
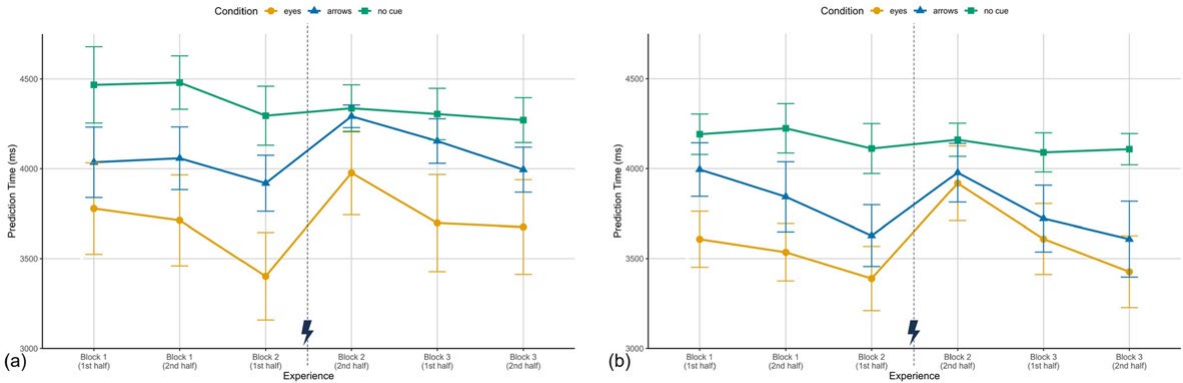
Prediction time in Study I (**a**) and Study II (**b**) for selecting the correct targeted square in milliseconds over the three experimental blocks, each split in the first six trials and the last six trials, for the conditions eyes, arrows and no cue. Depicted are means and standard errors. The lightning bolt icon marks the occurrence of cue error trials.

Prediction time (the duration in milliseconds from trial onset until participants selected the correct movement target on the tablet) is displayed in Fig. 4 (a) for Study I and (b) for Study II. Descriptively, the prediction time patterns closely mirror the results of the time to first target fixation: participants in the eyes condition were consistently the fastest across studies (Study I: $M_{E} = 3707.28$ ms, $SD = 906.76$ ms; Study II: $M_{E} = 3580.87$ ms, $SD = 610.04$ms), followed by the arrows condition (Study I: $M_{A} = 4075.72$ ms, $SD = 457.76$ms; Study II: $M_{A} = 3795.35$ ms, $SD = 635.02$ms). Participants without predictive cues were consistently the slowest (Study I: $M_{Nc} = 4359.02$ ms, $SD = 543.36$ ms; Study II: $M_{Nc} = 4147.46$ ms, $SD = 398.99$ ms). Both figures also show a rise in prediction times after the cue errors in block 2. To examine whether these descriptive patterns were statistically supported, we conducted mixed ANOVAs for each block.

In Study I in block 1, the condition effect narrowly missed conventional significance, $F(2,39) = 3.22$, $p = .051$, $\eta ^2_{p} = .14$, though with a moderately large effect size. In Study II the condition effect got significant showing an even stronger effect ($F(2,39) = 4.62$, $p = .016$, $\eta ^2_{p} = .19$). To allow proper comparison between Study I and II, post hoc tests were performed for both studies. Bonferroni corrected post hoc tests revealed for both studies significantly faster predictions in the eyes than the no cue condition (Study I: $t(39) = -2.53$, $p = .047$, $d = -.81$; Study II: $t(39) = -3.04$, $p = .013$. $d = -.97$), while differences between eyes and arrows (Study I: $t(39) = -1.05$, $p = .905$, $d = -.34$: Study II: $t(39) = -1.66$, $p = .316$, $d = -.53$) and between arrows and no cues (Study I: $t(39) = -1.48$, $p = .441$, $d = -.47$; Study II: $t(39) = -1.38$, $p = .529$, $d = .44$) were not significant. Neither the experience effect nor the interaction reached significance (Study I: all $F < 1$; Study II: experience: $F(1,39) = 1.93$, $p = .172$, $\eta ^2_{p} = .05$; interaction: $F(2,39) = 1.37$, $p = .267$, $\eta ^2_{p} = .07$).

For the second block, where the error occurred mid-block, we found in Study I significant effects of condition, $F(2,39) = 3.62$, $p = .004$, $\eta ^2_{p} = .16$, experience, $F(1,39) = 27.92$, $p < .001$, $\eta ^2_{p} = .42$, and their interaction, $F(2,39) = 6.22$, $p = .005$, $\eta ^2_{p} = .24$. Study II replicated the interaction, $F(2,39) = 4.91$, $p = .013$, $\eta ^2_{p} = .20$, and the experience effect, $F(1,39) = 23.92$, $p < .001$, $\eta ^2_{p} = .38$, but not the main effect of condition, $F(2,39) = 2.60$, $p = .087$, $\eta ^2_{p} = .12$. Bonferroni corrected post hoc tests showed that in the first half of block 2, participants in the eyes condition were faster than those without cues (Study I: $t(39) = -3.29$, $p = .006$, $d = -1.05$; Study II: $t(39) = -3.12$, $p = .010$, $d = -.99$) while the difference between arrows and no cues did not get significant (Study I: $t(39) = -1.39$, $p = .521$, $d = -.45$; Study II: $t(39) = -2.09$, $p = .130$. $d = -.67$). Similar to the results of the time to first target fixation, the difference between eyes and no cues disappeared in the second half (Study I: $t(39) = -1.61$, $p = .350$, $d = -.52$; Study II: $t(39) = -1.06$, $p = .089$, $d = -.34$) as prediction times slowed down significantly after the cue error for both predictive cue conditions (Eyes Study I: $t(39) = -5.32$, $p < .001$, $d = -.84$, Study II: $t(39) = -4.83$, $p < .001$, $d = -.76$; Arrows Study I: $t(39) = -3.50$, $p = .001$, $d = -.55$; Study II: $t(39) = -3.19$, $p = .003$, $d = -.50$). Prediction times in the no cue condition remained stable, as expected, since there was no error (Study I: $t(39) = -0.38$, $p = .704$, $d = -.06$; Study II: $t(39) = -0.45$, $p = .657$, $d = -.07$).

Block 3 descriptively mirrored the pattern of block 1 with eyes being the fastest condition and no cues being the slowest condition. However, the condition effect did not reach significance, while still showing moderate to large effect sizes (Study I: $F(2,39) = 2.72$, $p = .079$, $\eta ^2_{p} = .12$; Study II: $F(2,39) = 3.21$, $p = .051$, $\eta ^2_{p} = .14$). Likewise, there was no significant interaction (Study I: $F(2,39) = 1.90$, $p = .164$, $\eta ^2_{p} = .09$; Study II: $F(2,39) = 2.44$, $p = .100$, $\eta ^2_{p} = .11$). Instead, a significant effect of experience emerged (Study I: $F(1,39) = 5.03$, $p = .031$, $\eta ^2_{p} = .11$; Study II: $F(1,39) = 6.09$, $p = .018$, $\eta ^2_{p} = .14$), indicating faster predictions and potential re-engagement with the cues over the course of block 3.

A mini meta-analytical approach following^51^ was again applied exploratory to aggregate the effects of cue type on prediction time across both studies (see Table 3). As for the time to first target fixation, the data were combined into pre-error and post-error phases to examine cue effects at a more general level. Overall, cueing effects relative to the no cue condition were larger in the pre-error than in the post-error phase. This pattern was evident for both comparisons, eyes versus no cue and arrows versus no cue. The eyes versus no cue comparison showed moderate effects pre-error ($r = .43$) and smaller effects post-error ($r = .28$), which were significant in both phases ($Z = 3.96$, $p < .001$; $Z = 2.60$, $p = .009$). In contrast, the arrows versus no cue comparison yielded smaller effects overall ($r = .25$ pre-error; $r = .14$ post-error), reaching significance only in the pre-error phase ($Z = 2.25$, $p = .025$) but not post-error ($Z = 1.31$, $p = .190$). Interestingly, the eyes versus arrows comparison showed effects of similar magnitude ($r = .22$ pre-error; $r = .15$ post-error), with a significant difference pre-error ($Z = 2.02$, $p = .044$) but not post-error ($Z = 1.35$, $p = .176$). Taken together, these results closely mirror the findings from the visual attention allocation. Specifically, they suggest that eyes provide a stronger benefit than arrows relative to the no cue condition. Furthermore, cueing benefits are generally more pronounced prior to the occurrence of an error and both cue types appear to be affected by the error.

**Table 3:** Weighted aggregated prediction time effects pre- and post-error.

	Pre-error			Post-error		
	Eyes–No Cue	Arrows–No Cue	Eyes–Arrows	Eyes–No Cue	Arrows–No Cue	Eyes–Arrows
$r_{Study I}$	0.422	0.224	0.230	0.298	0.081	0.225
$r_{Study II}$	0.442	0.267	0.210	0.268	0.205	0.070
$M_{rz}$	0.462	0.251	0.223	0.291	0.144	0.150
$M_{r}$	0.432	0.245	0.220	0.283	0.143	0.149
Combined *Z*	3.96***	2.25*	2.02*	2.60**	1.31	1.35

*Note*. $r_{Study I}$ and $r_{Study II}$ denote effect sizes (Pearson’s *r*) derived from the pairwise post hoc comparisons within each study. $M_{rz}$ represents the weighted mean of Fisher’s *z*-transformed correlations, and $M_{r}$ the corresponding back-transformed mean correlation. Combined *Z* values were calculated using Stouffer’s method across the two studies. Pre-error comprises block 1 and block 2 (pre-error), whereas post-error comprises block 2 (post-error) and block 3. Positive values indicate faster responses (i.e., lower prediction times) in the first-mentioned condition of each comparison. *$p < .05$. **$p < .01$. ***$p < .001$.

Apart from the developments of prediction time over the experimental blocks, across both studies, prediction time correlated negatively with fixations on the robotic display in the cue conditions, indicating that greater cue usage was associated with faster predictions (Study I: $r = -.55$, $t(26) = -3.36$, $p = .002$; Study II: $r = -.62$, $t(26) = -4.01$, $p < .001$).

In addition to the prediction time, the prediction accuracy was also of interest. Prediction accuracy was high across conditions and blocks (Study I: $M_{Nc} = 0.95$, $SD = .07$; $M_{A} = 0.95$, $SD = .07$; $M_{E} = 0.88$, $SD = .14$; Study II: $M_{Nc} = 0.94$, $SD = .10$; $M_{A} = 0.95$, $SD = .06$; $M_{E} = 0.95$, $SD = .04$). Because no speed–accuracy trade-off was expected, we consequently expected no differences across conditions, and performed Bayesian mixed ANOVAs. In block 1, there was weak to moderate evidence for the null hypothesis of no condition differences (Study I: $BF_{01} = 2.24$; Study II: $BF_{01} = 4.70$). In block 2, evidence for the null remained weak but favored the model without condition (Study I: $BF_{01} = 1.97$; Study II: $BF_{01} = 2.28$). In block 3, Study I showed weak evidence against the null ($BF_{01} = 0.65$), whereas Study II showed moderate evidence for it ($BF_{01} = 6.08$), making the null hypothesis 6.8 times more likely than the alternative.

#### Performance in memory task

Performance in the memory task was assessed using a ratio of hits to false alarms (range: $-1$ to $+1$). Descriptively, participants in the cue conditions performed best. In Study I, performance was highest in the eyes condition ($M_{E} = 0.86$, $SD = 0.11$), followed by arrows ($M_{A} = 0.78$, $SD = 0.11$), while the no cue condition showed the lowest performance values ($M_{Nc} = 0.77$, $SD = 0.14$). Study II showed a similar pattern, with high performance in the eyes ($M_{E} = 0.83$, $SD = 0.14$) and arrows conditions ($M_{A} = 0.86$, $SD = 0.08$), and slightly lower performance without cues ($M_{No Cue} = 0.79$, $SD = 0.11$).

The inferential results for both studies are summarized in Table 4. In Study I, the only significant effect was a performance improvement within block 3 ($F(1,39) = 4.1$, $p = .049$, $\eta ^2_{p} = .10$). In Study II, the ANOVA for block 1 revealed a significant experience ($F(1,39) = 17.25$, $p < .001$, $\eta ^2_{p} = .31$) and interaction effect ($F(2,39) = 5.67$, $p = .007$, $\eta ^2_{p} = .23$). Post hoc tests showed that performance improved from the first to the second half of block 1 in the arrows condition ($t(38) = -4.76$, $p < .001$,$d = -.75$) as well as the condition without cues ($t(38) = -2.44$, $p = .019$, $d = .39$), but not in the eyes condition ($t(38) = 0.01$, $p = .995$, $d < .01$). Study II also revealed a significant performance improvement in block 2 ($F(1,39) = 8.95$, $p = .005$, $\eta _p^2 = .19$). No significant effects were observed in block 3.

Across both studies, performance in the memory task was significantly negatively correlated with prediction time, indicating that faster identification of the robot’s target was associated with better memory task performance (Study I: $r = -.62$, $t(40) = -5.02$, $p < .001$; Study II: $r = -.47$, $t(40) = -3.36$, $p = .002$).

**Table 4:** Memory task performance.

B	Condition effect	Experience effect	Interaction effect
1	I: $F(2,39) = 1.80$, $p = .18$, $\eta ^2_p = .08$ II: $F(2,39) = 0.44$, $p = .65$, $\eta ^2_p = .02$	I: $F(1,39) = 0.32$, $p = .57$, $\eta ^2_p < .01$ II: $F(1,39) = 17.25$, $p < .01$, $\eta ^2_p = .31$	I: $F(2,39) = 0.23$, $p = .80$, $\eta ^2_p = .01$ II: $F(2,39) = 5.67$, $p = .01$, $\eta ^2_p = .23$
2	I: $F(2,39) = 1.15$, $p = .33$, $\eta ^2_p = .06$ II: $F(2,39) = 2.27$, $p = .12$, $\eta ^2_p = .10$	I: $F(1,39) = 0.56$, $p = .46$, $\eta ^2_p = .01$ II: $F(1,39) = 8.95$, $p = .01$, $\eta ^2_p = .19$	I: $F(2,39) = 0.62$, $p = .54$, $\eta ^2_p = .03$ II: $F(2,39) = 1.38$, $p = .26$, $\eta ^2_p = .07$
3	I: $F(2,39) = 2.52$, $p = .09$, $\eta ^2_p = .11$ II: $F(2,39) = 1.87$, $p = .17$, $\eta ^2_p = .09$	I: $F(1,39) = 4.13$, $p = .05$, $\eta ^2_p = .10$ II: $F(1,39) = 1.63$, $p = .21$, $\eta ^2_p = .04$	I: $F(2,39) = 0.11$, $p = .90$, $\eta ^2_p < .01$ II: $F(2,39) = 2.18$, $p = .13$, $\eta ^2_p = .10$

*Note*. ANOVA test statistics for the memory task performance for block (B) 1, block 2 and block 3 in Study I and Study II.

### Trust

The two items of the used trust questionnaire^46^ were averaged to a mean trust score (scale 1–5) with higher values reflecting higher subjective trust. Ratings were collected at four points during the experiment: before interaction ($t_{0}$), after error-free interaction ($t_{1}$), after the predictive cue error ($t_{2}$), and after repeated error-free interaction ($t_{3}$).

**Fig. 5:**
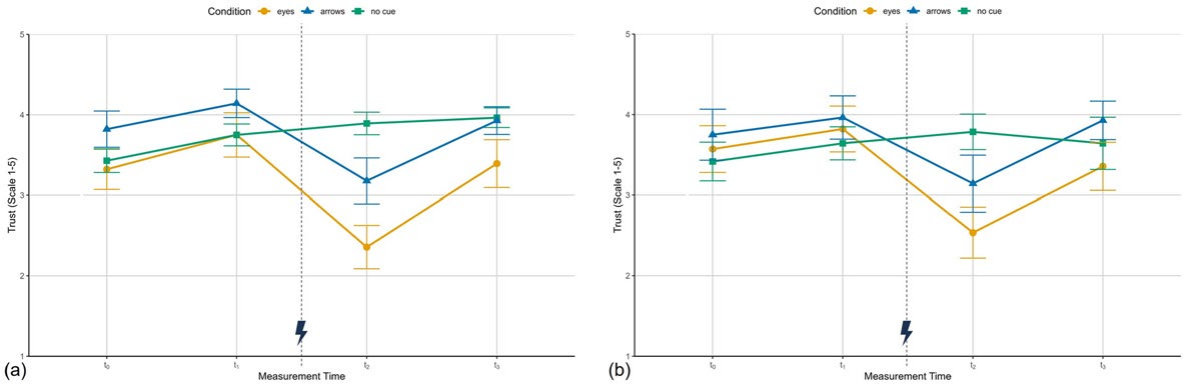
Subjective trust in Study I (**a**) and Study II (**b**) for the three conditions across the initial trust measurement ($t_{0}$), trust after error-free interaction in block 1 ($t_{1}$), trust after failure experience in block 2 ($t_{2}$) and trust after error-free interaction in block 3 ($t_{3}$). Depicted are means and standard errors. The lightning bolt icon marks the occurrence of cue error trials.

Descriptively (see Fig. 5), trust increased most consistently in the no cue condition across both studies, which is expected because this condition involved no errors. In contrast, the cue conditions showed a rise from $t_{0}$ to $t_{1}$, a decline after the predictive-cue error ($t_{2}$), and a subsequent recovery at $t_{3}$. Across studies and measurement times, average trust was lowest in the eyes condition (Study I: $M_{E} = 3.21$, $SD = 0.72$; Study II: $M_{E} = 3.32$, $SD = 0.84$) compared to the arrows (Study I: $M_{A} = 3.77$, $SD = 0.62$; Study II: $M_{A} = 3.70$, $SD = 0.89$) and no cue conditions (Study I: $M_{No Cue} = 3.76$, $SD = 0.41$; Study II: $M_{No Cue} = 3.63$, $SD = 0.79$).

In Study I, a 3x4 mixed ANOVA revealed significant effects of condition ($F(2,39) = 4.10$, $p = .024$, $\eta ^2_{p} = .17$), time ($F(2.74,106.75) = 10.85$, $p < .001$, $\eta ^2_{p} = .22$; Greenhouse–Geisser corrected), and their interaction ($F(5.47,106.75) = 4.53$, $p < .001$, $\eta ^2_{p} = .19$). All post hoc *p*-values were Bonferroni corrected for four tests.

To test the first hypothesis of trust formation during error-free interaction as well as the expectation of a stronger formation in conditions with predictive cues, a post hoc 3x2 mixed ANOVA for the trust formation phase from $t_{0}$ to $t_{1}$ was conducted. The ANOVA indicated an increase in trust over time, which, however, just missed significance, ($F(1,39) = 6.32$, $p = .065$, $\eta ^2_{p} = .14$), while still showing a large effect. There were no effects of condition ($F(2,39) = 2.09$, $p = .547$, $\eta ^2_{p} = .10$) or interaction ($F(2,39) = 0.06$, $p> .999$, $\eta ^2_{p} < .01$), indicating that trust did not form more strongly in the cue conditions. For the second hypothesis expecting a trust decrease after failure experience, a 3x2 ANOVA for the dissolution phase ($t_{1}$ to $t_{2}$) was performed. It showed a substantial condition effect that, however, just missed the conventional level of significance ($F(2,39) = 4.56$, $p = .066$, $\eta ^2_{p} = .19$), and comparatively larger significant effects of time ($F(1,39) = 29.22$, $p < .001$, $\eta ^2_{p} = .43$) and interaction ($F(2,39) = 11.23$, $p < .001$, $\eta ^2_{p} = .37$). Trust decreased after the cue error in the eyes ($t_{1}$: $M_{E} = 3.75$, $SD = 1.03$; $t_{2}$: $M_{E} = 2.36$, $SD = 1.01$) and arrows conditions ($t_{1}$: $M_{A} = 4.14$, $SD = 0.66$; $t_{2}$: $M_{A} = 3.18$, $SD = 1.07$), but slightly increased in the no cue condition ($t_{1}$: $M_{Nc} = 3.75$, $SD = 0.51$; $t_{2}$: $M_{Nc} = 3.89$, $SD = 0.53$). For the last hypothesis that trust increases again with error-free interaction after failure experience, another 3x2 ANOVA from $t_{2}$ to $t_{3}$ was conducted showing significant condition ($F(2,39) = 6.83$, $p = .011$, $\eta ^2_{p} = .26$), time ($F(1,39) = 30.57$, $p < .001$, $\eta ^2_{p} = .44$) and interaction effects ($F(2,39) = 6.52$, $p = .014$, $\eta ^2_{p} = .25$). Trust recovered in the eyes ($t_{2}$: $M_{E} = 2.36$, $SD = 1.01$; $t_{3}$: $M_{E} = 3.39$, $SD = 1.11$) and arrows conditions ($t_{2}$: $M_{A} = 3.18$, $SD = 1.07$; $t_{3}$: $M_{A} = 3.93$, $SD = 0.65$), but showed little change in the no cue condition ($t_{2}$: $M_{Nc} = 3.89$, $SD = 0.53$; $t_{3}$: $M_{Nc} = 3.96$, $SD = 0.46$). In terms of whether trust in the predictive cue conditions remained below the original level, a post hoc comparison of $t_{1}$ and $t_{3}$ for the eyes and arrows condition revealed that in both conditions, the trust level between $t_{1}$ and $t_{3}$ did not differ significantly (Eyes: $t(39) = 1.48$, $p = .148$, $d = .23$; Arrows: $t(39) = 0.89$, $p = .381$, $d = .14$).

In Study II, the 3x4 mixed ANOVA showed significant effects of time ($F(2.22,82.19) = 5.14$, $p = .006$, $\eta ^2_{p} = .12$) and interaction ($F(4.44,82.19) = 2.95$, $p = .021$, $\eta ^2_{p} = .14$), but no condition effect ($F < 1$). Compared to Study I, these effect sizes tended to be smaller for the main effects, while the interaction effect remained in a similar range. Post hoc *p*-values were Bonferroni corrected for four tests. Trust formation from $t_{0}$ to $t_{1}$ revealed no significant effects of condition ($F < 1$), time ($F(1,37) = 2.83$, $p = .404$, $\eta ^2_{p} = .07$), or interaction ($F < 1$). For the trust dissolution phase the results of the 3x2 ANOVA ($t_{1}$ to $t_{2}$) revealed no effect of condition ($F(2,39) = 1.43$, $p> .999$, $\eta ^2_{p} = .07$), but significant substantial effects of time ($F(1,39) = 12.68$, $p = .004$, $\eta ^2_{p} = .25$) and interaction ($F(2,37) = 5.24$, $p = .039$, $\eta ^2_{p} = .21$). As can be seen in Fig. 5 (b) trust decreased from $t_{1}$ to $t_{2}$ in the eyes ($t_{1}$: $M_{E} = 3.82$, $SD = 1.07$; $t_{2}$: $M_{E} = 2.54$, $SD = 1.18$) and arrows conditions ($t_{1}$: $M_{A} = 3.96$, $SD = 1.01$; $t_{2}$: $M_{A} = 3.14$, $SD = 1.32$), but increased slightly in the no cue condition ($t_{1}$: $M_{Nc} = 3.64$, $SD = 0.77$; $t_{2}$: $M_{Nc} = 3.79$, $SD = 0.83$). For the trust restoration phase another 3x2 ANOVA from $t_{2}$ to $t_{3}$ was conducted, which showed significant time ($F(1,39) = 12.44$, $p = .004$, $\eta ^2_{p} = .24$) and interaction effects ($F(2,39) = 5.20$, $p = .040$, $\eta ^2_{p} = .21$), but no condition effect ($F(2,39) = 2.20$, $p = .495$, $\eta ^2_{p} = .10$). Similar to Study I, the significant interaction supports the claim of increasing trust ratings in the eyes ($t_{2}$: $M_{E} = 2.54$, $SD = 1.18$; $t_{3}$: $M_{E} = 3.36$, $SD = 1.12$) and arrows condition ($t_{2}$: $M_{A} = 3.14$, $SD = 1.32$; $t_{3}$: $M_{A} = 3.93$, $SD = 0.90$), but not in the condition without cues ($t_{2}$: $M_{Nc} = 3.79$, $SD = 0.83$; $t_{3}$: $M_{Nc} = 3.64$, $SD = 1.22$). A post hoc comparison of $t_{1}$ and $t_{3}$ further showed that trust was again fully restored in both conditions (eyes: $t(37) = 2.04$, $p = .195$, $d = -.55$; arrows: $t(37) = 0.16$, $p> .99$, $d = .03$).

### Workload

Workload was assessed after each experimental block using the NASA-TLX. Ratings were provided on a 21-point scale ranging from very low (0) to very high (100). For the primary analysis, the six subscales were averaged to compute an overall workload score. Because we did not expect differences between conditions, we conducted a Bayesian ANOVA on this composite score. However, for transparency, separate Bayesian ANOVAs for each NASA-TLX subscale are reported on the OSF.

Across all measurement times in Study I, workload was rated on average with $M_{E} = 62.80$ ($SD = 10.47$) in the eyes condition (Study II: $M_{E} = 56.53$, $SD = 9.95$), $M_{A} = 59.25$ ($SD = 10.05$) in the arrows condition (Study II: $M_{A} = 59.48$, $SD = 6.90$) and $M_{Nc} = 55.48$ ($SD = 14.80$) in the condition without cues (Study II: $M_{Nc} = 66.53$, $SD = 9.98$). See Table 5 for detailed means and standard deviations per measurement time.

**Table 5:** Means and standard deviations for the NASA-TLX workload measure in Study I and Study II.

	Eyes *M(SD)*	Arrows *M(SD)*	No cue *M(SD)*
$t_1$	I: 63.15 (10.93) II: 61.13 (10.59)	I: 60.12 (15.44) II: 63.93 (10.66)	I: 58.39 (15.66) II: 68.87 (8.32)
$t_2$	I: 65.12 (11.10) II: 55.89 (11.01)	I: 60.24 (11.17) II: 60.83 (5.98)	I: 52.44 (15.56) II: 66.73 (11.78)
$t_3$	I: 60.12 (12.03) II: 52.56 (15.03)	I: 57.38 (9.43) II: 53.69 (11.36)	I: 55.60 (17.97) II: 63.98 (14.01)

*Note*. Measurement times are $t_{1}$ (after failure-free interaction in block 1), $t_{2}$ (after interaction with cue failure in block 2), and $t_{3}$ (after failure-free interaction in block 3).

To evaluate evidence for the null hypothesis that workload does not differ between conditions, a Bayesian mixed ANOVA compared a model including condition to a model including only time and participant. In the first study, results indicated $BF_{10} = 0.613$ (i.e., $BF_{01} = 1.63$), meaning the data were about 1.6 times more likely under the model without condition, which is weak evidence for no condition differences. In contrast, adding condition in Study II improved model fit ($BF_{10} = 3.69$; $BF_{01} = 0.27$), providing moderate evidence that workload differed between conditions. In addition to the NASA-TLX, a self-developed single-item workload measure was included in the second study. Bayesian model comparison indicated that adding condition did not improve the model ($BF_{10} = 0.82$; $BF_{01} = 1.23$) offering weak evidence for the absence of a condition effect. To examine whether the single item captured a construct similar to the NASA-TLX, we computed correlations between the two measures. The correlation between the NASA-TLX overall score and the single item was high ($r = .70$; $t(40) = 6.84$, $p < .001$). Additional correlations with NASA-TLX subscales at each measurement time are available on the OSF. These correlations varied widely across scales and time points. For example, physical demand consistently showed small correlations (all $r < .26$), while mental demand showed moderate to high correlations (e.g., $r_{t1} = .49$, $r_{t2} = .57$, $r_{t3} = .57$).

### Exploratory eye tracking analysis

#### Mean number of fixations in AOIs

The analysis focused on the robot’s display (showing predictive cues or remaining black) and the robotic arm (moving toward the target). Furthermore, only the results for block 2 are reported as they are of special interest due to potential shifts of attention allocation after the cue error.

**Fig. 6:**
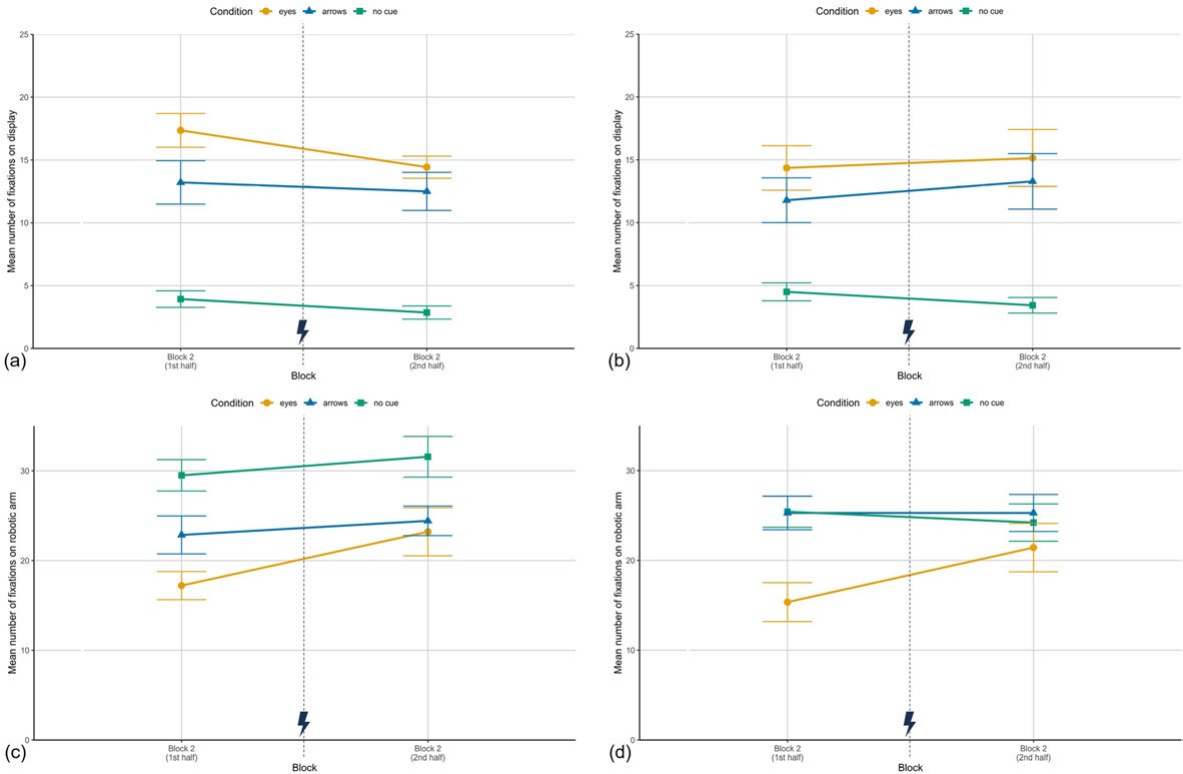
Mean number of fixations. (**a**) Study I: fixations on the AOI display per six trials in the first and the last six trials of block 2, for the three conditions eyes, arrows and no cue. (**b**) Study II: fixations on the AOI display. (**c**) Study I: Fixations on the AOI robotic arm per six trials in block 2. (**d**) Study II: Fixations on the AOI robotic arm. Depicted are means and standard errors. The lightning bolt icon marks the occurrence of cue error trials.

For the display fixations, the manipulation check already revealed significantly more fixations on the display in the predictive cue conditions compared to the no cue condition in the first block. As shown in Fig. 6 (a, b), this pattern continued in block 2. Per six trials, participants in the eyes condition fixated the display 15.89 times ($SD = 3.03$), in the arrows condition 12.86 times ($SD = 5.26$) and 3.39 times ($SD = 2.07$) in the no cues condition in Study I (Study II: $M_{E} = 14.75$, $SD = 7.07$; $M_{A} = 12.54$, $SD = 7.20$; $M_{Nc} = 3.96$, $SD = 2.30$). The ANOVA revealed a strong significant main effect of cue condition (Study I: $F(2,38) = 43.43$, $p < .001$, $\eta ^2_{p} = .69$; Study II: $F(2,39) = 12.71$, $p < .001$, $\eta ^2_{p} = .39$). Post hoc tests confirmed more display fixations in the eyes condition compared to no cues (Study I: $t(39) = -8.93$, $p < .001$, $d = -2.86$; Study II: $t(39) = -4.77$, $p < .001$, $d = -1.53$) as well as the arrows condition compared to no cues (Study I: $t(39) = -6.77$, $p < .001$, $d = -2.17$; Study II: $t(39) = 3.79$, $p = .002$, $d = 1.21$), while eyes and arrows did not differ significantly (Study I: $t(39) = 2.17$, $p = .109$, $d = .70$; Study II: $t(39) = -0.98$, $p = .999$, $d = -.31$). In Study I, there was also an experience effect in block 2 ($F(1,39) = 4.12$, $p = .049$, $\eta ^2_p = .10$), indicating a decrease in display fixations after the error; Study II showed no such effect ($F < 1$). No interaction effects were found (Study I: $F < 1$; Study II: $F(2,39) = 1.40$, $p = .259$, $\eta ^2_p = .07$).

For the robotic arm (Fig. 6 (c, d)), fixation counts averaged over block 2 were generally higher than for the display. In Study I, participants in the no cue condition fixated the arm 30.54 ($SD = 6.96$) times per six trials (Study II: $M_{Nc} = 24.82$, $SD = 6.76$), in the arrows condition 23.64 ($SD = 6.42$) times (Study II: $M_{A} = 25.29$, $SD = 6.74$), and in the eyes condition 20.21 ($SD = 7.55$) times (Study II: $M_{E} = 18.39$, $SD = 8.61$).

The ANOVA for block 2 in Study I showed a significant effect of condition ($F(2,39) = 7.91$, $p = .001$, $\eta ^2_{p} = .29$) with more arm fixations in the no cue condition than in the eyes ($t(39) = 3.91$, $p = .001$, $d = 1.25$) and arrows condition ($t(39) = 2.61$, $p = .039$, $d = .84$). There was no difference between eyes and arrows ($t(39) = -1.30$, $p = .607$, $d = -.42$). There was also an experience effect ($F(1,39) = 11.49$, $p = .002$, $\eta ^2_p = .23$), reflecting increased arm fixations after the error. There was no interaction between cue condition and experience ($F(2,39) = 2.18$, $p = .126$, $\eta ^2_{p} = .10$). In Study II, we found significant condition and interaction effects, while the effect of experience missed significance (Condition: $F(2,39) = 3.77$, $p = .032$, $\eta ^2_{p} = .16$; experience: $F(1,39) = 3.50$, $p = .069$, $\eta ^2_{p} = .08$; interaction: $F(2,39) = 6.78$, $p = .003$, $\eta ^2_{p} = .26$). Post hoc tests indicated a significant increase of arm fixations in the eyes condition ($t(39) = -4.05$, $p < .001$, $d = -.64$) after the error, diminishing a present pre-error difference between eyes and no cue ($t(39) = 3.69$, $p = .002$, $d = 1.18$) as well as eyes and arrows ($t(39) = 3.64$, $p = .002$, $d = 1.17$), which wasn’t present anymore after error occurrence (eyes vs. no cue: $t(39) = 0.86$, $p> .999$, $d = .28$; eyes vs. arrows: $t(39) = 1.19$, $p = .726$, $d = .38$). There was no difference between arrows and no cue, neither in pre- ($t(39) = -0.05$, $p> .999$, $d = -.02$) nor in post-error trials ($t(39) = 0.33$, $p> .999$, $d = .11$) as there was also no significant change for both conditions over time (arrows: $t(39) < 0.01$, $p> .999$, $d < .01$; no cue: $t(39) = 0.81$, $p = .423$, $d = .13$).

#### Mean fixation duration in AOIs

**Fig. 7:**
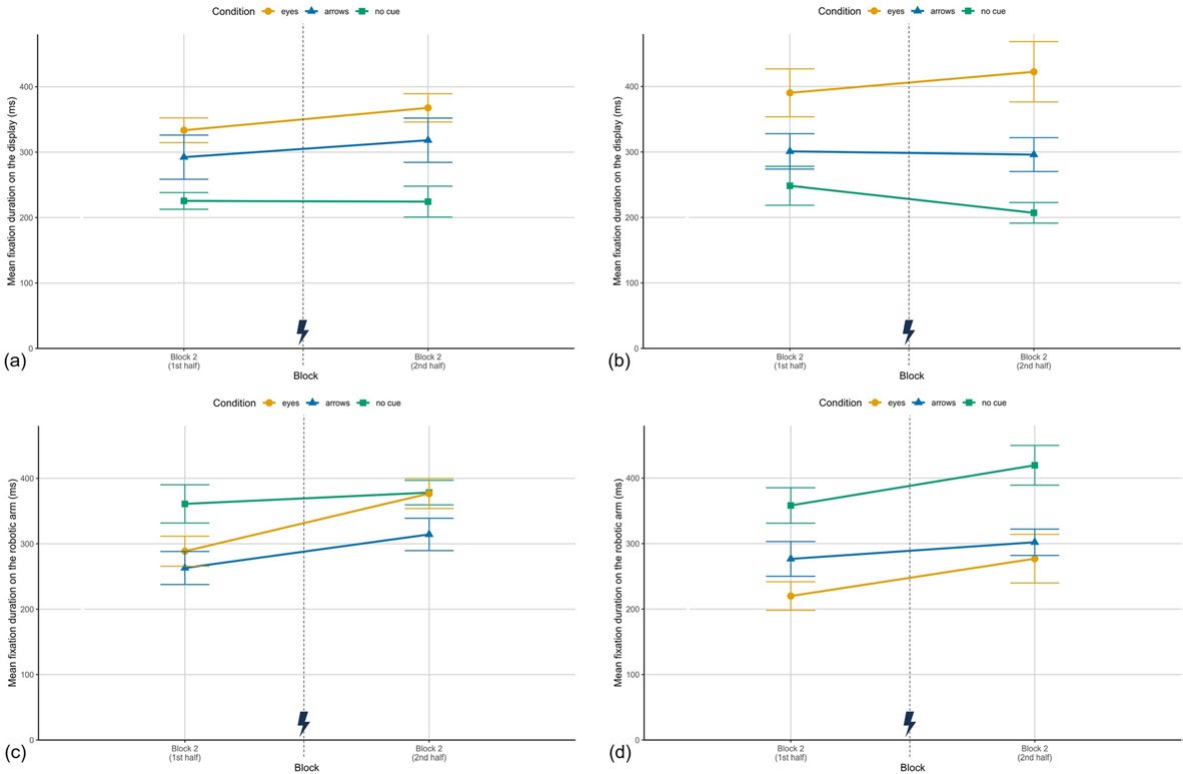
Mean fixation duration. (**a**) Study I: Fixation duration in ms on the AOI display per six trials in the first and the last six trials of block 2, for the three conditions eyes, arrows and no cue. (**b**) Study II: Fixation duration in ms on the AOI display. (**c**) Study I: Fixation duration in ms on the AOI robotic arm per six trials in block 2. (**d**) Study II: Fixation duration in ms on the AOI robotic arm. Depicted are means and standard errors. The lightning bolt icon marks the occurrence of cue error trials.

The mean fixation duration was also analyzed for the robot’s display and the robotic arm in block 2 (see Fig. 7).

Display fixations were shortest in the no cue condition ($M_{Nc} = 222.28$ ms, $SD = 56.97$ ms; Study II: $M_{Nc} = 226.75$ ms, $SD = 77.35$ ms), slightly longer with arrows (Study I: $M_{A} = 305.28$ ms, $SD = 119.69$ ms; Study II: $M_{A} = 298.44$ ms, $SD = 90.77$ ms), and longest with eyes (Study I: $M_{E} = 350.59$ ms, $SD = 70.46$ ms; Study II: $M_{E} = 406.41$ ms, $SD = 141.03$ ms). In the second block of Study I, the condition effect was significant ($F(2,37)=6.26$, $p=.005$, $\eta _p^2=0.25$). Fixations were longer in the eyes condition than in the no cue condition ($t(37)=-3.52$, $p=.004$, $d = -1.16$), with no differences between arrows and no cues ($t(37)=-2.22$, $p=.099$, $d = -.73$) or between arrows and eyes ($t(37)=1.35$, $p=.552$, $d = .44$). Study II replicated the condition effect ($F(2,38)=9.21$, $p<.001$, $\eta _p^2=0.33$), with post hoc tests showing longer display fixations in the eyes condition than the condition without cues ($t(38) = -4.24$, $p < .001$, $d = -1.38$) and, different to Study I, also significantly longer fixations than in the arrows condition ($t(38) = 2.65$, $p = .035$, $d = .86$). There was no difference in fixation duration between participants in the arrows and no cue condition ($t(38) = -1.64$, $p = .327$, $d = -.53$). Experience and interaction effects were non-significant in both studies (Experience Study I and II: $F < 1$; Interaction Study I: $F(2,37) = 1.16$, $p = .323$, $\eta ^2_{p} = .06$; Study II: $F(2,38) = 1.99$, $p = .151$, $\eta ^2_{p} = .09$).

For the robotic arm (see Fig. 7 (c) and (d)), the overall fixation durations are quite similar to those on the display, but with a different ordering. Fixations were longest in the no cues condition in both studies (Study I: $M_{Nc} = 369.58$ms, $SD = 83.57$ms; Study II: $M_{Nc} = 388.91$ms, $SD = 99.72$ms). In Study I, eyes produced intermediate fixation durations ($M_{E} = 332.64$ ms, $SD = 77.60$ ms) and arrows the shortest ($M_{A} = 288.61$ ms, $SD = 90.37$ ms). In Study II, eyes produced the shortest fixations ($M_{E} = 248.43$ ms, $SD = 107.61$ ms), followed by arrows ($M_{A} = 289.32$ ms, $SD=83.99$ ms). The ANOVA for block 2 showed, apart from significant condition (Study I: $F(2,39) = 3.26$, $p = .049$, $\eta ^2_{p} = .14$; Study II: $F(1,39) = 7.67$, $p = .002$, $\eta ^2_{p} = .28$) and experience effects (Study I: $F(1,39) = 25.48$, $p < .001$, $\eta ^2_{p} = .40$; Study II: $F(1,39) = 19.48$, $p < .001$, $\eta ^2_{p} = .33$), also a significant interaction effect (Study I: $F(2,39) = 3.91$, $p = .028$, $\eta ^2_{p} = .17$), however only in the first study. In the second study, the interaction effect was not significant (Study II: $F(2,39) = 1.09$, $p = .348$, $\eta ^2_{p} = .05$). Post hoc tests for the interaction in Study I showed that pre-error fixation durations differed between no cues and arrows ($t(39)=2.67$, $p=.033$, $d = .85$), but not between no cues and eyes ($t(39)=1.97$, $p=.167$, $d = .63$) or eyes and arrows ($t(39)=0.70$, $p>.999$, $d = .22$). After the error, fixation durations increased significantly in the arrows ($t(39)=2.86$, $p=.007$, $d = .45$) and eyes conditions ($t(39)=-4.92$, $p<.001$, $d = -.78$), but not in the no cue condition ($t(39)=-0.96$, $p=.341$, $d = -.15$). Consequently, post-error comparisons showed no differences across conditions (No cue vs. arrows: $t(39) = 2.03$, $p = .147$, $d = .65$; no cue vs. eyes: $t(39) = 0.05$, $p> .999$, $d = .02$; eyes vs. arrows $t(39) = 1.98$, $p = .163$, $d = .63$). In Study II, post hoc tests for the condition effect showed overall significantly longer fixations on the robotic arm in the no cues condition compared with eyes ($t(39) = 3.81$, $p = .002$, $d = 1.22$) and arrows ($t(39) = 2.70$, $p = .031$, $d = .86$), with no difference between eyes and arrows ($t(39) = -1.11$, $p = .823$, $d = -.36$). Furthermore, the experience effect indicates significantly increased fixation durations after the error occurrence across conditions.

## Discussion

The overarching goal of this research was to advance our understanding of how predictive cues shape visual attention, behavioral performance, and trust in industrial human-robot interaction. Across two controlled laboratory studies, one of which constituted a direct replication, we investigated how gaze-like cues, arrows, and the absence of cues influence humans’ ability to predict a robot’s actions, particularly under conditions of instrumental uncertainty introduced by cue failures. Both studies closely followed the paradigm of our previous work^37^, while implementing several methodological refinements to address earlier limitations. In the present studies, participants interacted with an industrial CoBot (Sawyer) that displayed either gaze-like cues, arrows, or no cues on its screen. Participants were instructed to predict the robot’s upcoming movement target as quickly and accurately as possible. Before each arm movement, the predictive cues, either eyes or arrows, shifted toward the intended target, providing participants in those conditions with task-relevant information about the robot’s next action. After selecting the predicted target on a tablet, participants completed a brief memory and visual search task before proceeding to the next trial. To investigate how participants responded to erroneous predictive signals, we introduced deliberate cue errors in one of the three experimental blocks, during which the displayed cue indicated an incorrect target and therefore mismatched the robot’s actual movement. By combining eye-tracking, behavioral, and attitudinal measures with an improved experimental paradigm, the present work extends prior findings^37^ and provides deeper insights on how predictive cues support (and sometimes might impair) joint action with robots.

Our manipulation check confirmed that participants consistently directed their attention to the predictive cues, which was reflected both in the gaze data and in self-reported cue usage. This demonstrates that the adjustments implemented in the present studies, particularly the explicit instruction of the cue mechanism, effectively addressed the major limitation of limited cue use in our previous study^37^. As expected, most control variables (perceived anthropomorphism in appearance and movement, individual tendency to anthropomorphize robots, mind perception, and regulatory focus) did not show meaningful group differences, indicating that the experimental manipulation was not confounded by individual differences. The only notable exception was a significant difference in the perceived contextual anthropomorphism in Study II, where context was rated as least anthropomorphic in the condition without cues ($M_{Nc} = 22.20$, $SD = 15.40$) and more anthropomorphic in the cue conditions ($M_{E} = 39.12$, $SD = 18.01$; $M_{A} = 39.33$, $SD = 20.95$). Because this pattern was not replicated across studies or anthropomorphism dimensions, it should be interpreted with caution and warrants future replication.

Building on this confirmed cue engagement, the visual attention results provide insight into how predictive cues influenced attentional allocation during the task. Across both studies, participants in the eyes condition fixated the correct target faster than those without cues during error-free interaction as reflected in a large aggregated effect across studies ($M_r = .53$), indicating that gaze cues can act as attentional signals, rapidly orienting observers toward task-relevant spatial information. In contrast, arrows did not show consistent attentional benefits relative to the no cue condition at the single-study level. Only in Study II, during the pre-error phase of Block 2, did arrows lead to significantly faster target fixation than no cues, whereas at other time points no significant advantage was observed. However, the aggregated mini meta-analytic results indicate that arrows do yield reliable effects overall, with small effect sizes that are significant in both the pre-error ($M_r = .29$) and post-error phases ($M_r = .29$). At the same time, arrows rarely differed significantly from gaze cues at the single-study level, whereas the aggregated results indicate a moderate advantage of eyes over arrows in the pre-error phase ($M_r = .31$), which is reduced and no longer significant in the post-error phase ($M_r = .18$). Taken together, these findings suggest that both cue types can facilitate attentional orienting, but gaze cues produce more consistent and, particularly before the error, stronger effects across interaction phases.

Consistent with these attentional patterns, the behavioral results further demonstrate how predictive cues influenced action prediction performance. The present studies (in contrast to our previous study^37^) provide clear evidence that predictive cues, especially gaze-like cues, consistently facilitate action prediction when they function reliably. Across both studies, participants in the eyes condition predicted the robot’s target substantially faster than those in the no cue condition (up to 893 ms in Study I and 722 ms in Study II during the first half of block 2). This provides partial support for our first hypothesis: predictive cues, particularly gaze cues, can enhance prediction performance during error-free interaction. At the same time, we found no consistent evidence at the single-study level that gaze cues were significantly more effective than arrows, nor did arrows confer a measurable advantage over the no cue condition. However, the mini meta-analytic results provide a more differentiated picture: eyes ($M_r = .43$) showed larger benefits over no cues than arrows ($M_r = .25$), and the comparison between eyes and arrows also indicated an advantage for eyes in the pre-error phase ($M_r = .22$).

Crucially, the performance benefits of predictive cues proved fragile. Following a cue-action mismatch, prediction time increased in both cue conditions, eliminating prior advantages relative to the no cue condition and confirming our hypothesis that prediction performance would decline following cue errors. This suggests that participants reduced their compliance with the cues after the error, likely waiting for more confirmation from the robot’s arm movement before making a prediction. This interpretation is further supported by the eye-tracking data which revealed a corresponding shift in visual attention. In Study I, fixations on the cue display decreased after the error, while fixations on the robotic arm increased. In Study II, this shift of attention allocation toward the robot arm occurred specifically in the eyes condition. Furthermore, in both studies, the fixation duration on the robotic arm increased after the cue error. This attentional reallocation could indicate a strategic adaptation to increased instrumental uncertainty. When the predictive signal could no longer be trusted, participants relied more strongly on kinematic information from the robot’s arm movement to regain predictive certainty. In terms of the proposed framework assuming that predictive cues influence attention allocation, which may in turn support prediction, this pattern suggests that the relation between cue-based attentional orienting and behavioral prediction is not fixed, but may be recalibrated when the cue loses reliability. Under such conditions, attention appears to shift away from the cue and toward alternative information sources that may better support prediction. Importantly, this shift was temporary. In the post-error block, performance partially recovered, as reflected in increasingly faster predictions, suggesting that continued reliable cue behavior can gradually restore alignment with predictive signals. However, performance did not fully return to pre-error levels. Consistent with this pattern, the aggregated weighted effect sizes indicate that post-error effects are reduced overall but remain larger and significant for eyes ($M_r = .28$), whereas the effect for arrows is smaller and no longer significant ($M_r = .14$).

Taken together, these findings indicate that the methodological adjustments implemented in the present studies successfully increased cue usage and amplified predictive cue effects. The results align with prior research showing that predictive cues, especially gaze cues, can support action prediction in HRI^16,52^. Furthermore, they align with theories of joint action that emphasize prediction under uncertainty as a core coordination challenge. Predictive cues reduce uncertainty by providing information about an agent’s future actions. However, when these cues violate expectations, they not only lose their informational value but may actively increase uncertainty by conflicting with subsequent action evidence. From this perspective, gaze cues can be understood as efficient but potentially risky design elements: beneficial when reliable, yet, at least temporarily, costly when violated. More broadly, the results suggest that predictive cues may support coordination not simply because they are present, but because they can guide attention toward task-relevant information in a way that may facilitate action prediction as long as users continue to regard the cue as informative.

At the same time, the present findings should be interpreted in light of prior work reporting comparable or even stronger effects for non-biological cues such as arrows. For example, Gregory and Jackson^26^ observed similar or, in some cases, stronger cueing effects for arrows compared to gaze cues. Several factors likely contribute to the differences between their findings and ours. First, their study employed a classical computerized 2D spatial-cueing paradigm with static images, whereas our studies involved real-time interaction with a robot, introducing a more complex and dynamic context. Second, their gaze cues were pictures of a face and their arrows were simple symbolic cues, resulting in substantial differences in visual complexity, whereas in our studies both cue types were more comparable. Third, cues in Gregory and Jackson’s study were non-predictive, emphasizing the need to ignore them, whereas our cues were predictive and required active interpretation to guide behavior. Finally, their targets were presented on a 2D screen, while in our studies, cues on the robot’s display predicted targets in a 3D workspace. In this context, gaze cues may provide more salient depth information due to humans’ biologically attuned sensitivity to gaze direction, whereas arrows might be typically learned more as planar, left–right orientation signals. Together, these differences in cue characteristics, task demands, and spatial context may account for the divergent pattern of cueing effects observed across studies.

Following target prediction, participants completed a time-constrained memory and visual search task. Although participants in the eyes condition generally had more time available because they predicted the robot’s target more quickly, performance in this secondary task did not differ across conditions, contrary to our expectations. However, faster predictions were associated with better memory task performance across participants, suggesting that individual efficiency gains translated into better performance. The absence of condition effects likely reflects limited task sensitivity rather than a lack of functional relevance of prediction speed.

Beyond performance indicators, subjective trust was examined to assess how predictive cues and cue errors shape attitudinal responses. Within the proposed framework, trust was assumed to depend on whether the cue-based predictions are experienced as reliable or violated over time. Accordingly, any relation between cues and trust should be expected to be indirect and contingent on participants noticing and evaluating the cue over repeated interaction. Although trust ratings in the eyes condition were descriptively lower than in the arrows condition, this difference was not statistically tested as it was not part of our hypotheses. Importantly, both studies revealed the characteristic phases of trust dynamics: formation during error-free interaction, dissolution after error occurrence and restoration during subsequent reliable interaction. Inferential analyses confirmed trust dissolution and restoration in both studies, whereas trust formation was only descriptively observed. Therefore, the hypothesis of trust formation and stronger trust formation in the cue conditions had to be rejected. The hypothesis of a trust dissolution after failure experience was confirmed in both studies: after the cue error, trust decreased in the eyes and arrows condition, while slightly increasing in the condition without cues, where no error occurred. Additionally, trust restoration was evident in both studies with increasing trust in the eyes and arrows condition. Notably, these trust dynamics were absent in our previous study^37^, where low cue use and poor error detection likely prevented participants from registering the failure. In the present studies, higher cue engagement and higher error detection rates appear to have enabled the emergence of typical trust patterns, consistent with prior HRI work showing that errors reduce trust while error-free interaction builds trust^31,33,34,53^. Importantly, however, attitudinal trust does not necessarily translate into behavioral adaptation^54,55^. Several studies report weak associations between trust and behavioral outcomes^34,53,56^, and a recent review found only a small average correlation between trust and dependence behavior (*r* =.11)^55^. In contrast, our studies showed descriptively aligned patterns: decreases in trust coincided with slower prediction times, suggesting reduced compliance with predictive cues. This convergence should, however, be interpreted cautiously. It does not imply that trust directly caused changes in behavior, or vice versa. Rather, both may have been shaped in parallel by the same underlying experience with the predictive cues.

A broader conceptual issue concerns whether our paradigm adequately captures the uncertainty and vulnerability that are central to theoretical definitions of trust. Although these elements are often insufficiently represented in HRI paradigms^54^, one could argue that they were present here to some extent. Participants interacted in close physical proximity to a moving industrial robot, a context that can inherently evoke uncertainty. While approximately half of the participants reported prior experience with robots, this experience varied widely (e.g., from previous experiments, restaurant encounters, or academic courses), and it remains unclear to what extent such experience translates to interaction with an industrial collaborative robot. Furthermore, participants were depending on the robot to identify the movement target and to gain more time for the secondary tablet task. Dependability is, according to^54^, a key aspect for vulnerability and trust effects to occur. Consequently, participants may have experienced some uncertainty and vulnerability regarding the robot’s behavior. While both were not explicitly manipulated, the observed changes in both trust attitude and prediction behavior suggest that they may have been sufficiently salient to elicit meaningful trust dynamics in this context. Still, future studies could actively increase uncertainty and vulnerability (in an ethical manner) to further deepen the insights into trust attitude, trust dynamics and behavioral consequences of trust.

In addition to visual attention, performance and trust, we assessed subjective workload to examine whether predictive cues imposed any additional cognitive burden on participants. The Bayesian ANOVA results differed across the two studies. In Study I, the evidence leaned weakly toward the absence of condition effects, suggesting that predictive cues did not meaningfully affect workload. In Study II, the NASA-TLX indicated moderate evidence for workload differences between conditions; however, this pattern was not supported by the single-item workload measure, which instead provided weak evidence for no condition effect. Taken together, the findings remain inconclusive, and there is currently no consistent indication that predictive cues systematically increase or decrease perceived workload. We included the single-item workload measure to explore whether a simpler, unidimensional assessment could capture workload similarly to the multidimensional NASA-TLX. The strong correlation between the two measures ($r = .70$) suggests substantial overlap in the constructs they assess. However, correlations with individual NASA-TLX subscales varied widely, implying that the single-item measure may align more closely with some workload components (e.g., mental demand) than others (e.g., physical demand). Thus, while the single-item measure may be a pragmatic alternative, it does not fully replicate the multidimensional sensitivity of the NASA-TLX.

Although the present studies addressed several limitations of our previous work^37^ and substantially improved cue usage and error detection, several constraints remain. First, despite explicit instruction, a small number of participants still did not use the cues or failed to detect the cue error. In Study I, 4 of 28 participants reported never using the cues, in Study II, this was the case for 2 of 28 participants - substantially fewer than in our previous study (10 of 28). A similar pattern emerged for error detection: in Study I, 25 of 28 participants detected the cue error, in Study II, 22 of 28 detected it, compared with only 9 of 28 in the earlier study. These improvements indicate that instruction was effective, yet they also underline that even when users are made aware of cue mechanisms, time pressure or cognitive load may still hinder cue utilization. This has practical implications, as predictive cues may not always be attended to, and cue errors may still go unnoticed. Thus, in industrial applications, clear explanation, training, and repeated exposure may be necessary to ensure consistent cue use under demanding conditions. From a theoretical perspective, this also reinforces that the link between cue presentation and downstream behavioral or attitudinal effects is conditional on cue uptake, rather than automatic.

A second limitation concerns the eye-tracking methodology. As in the previous study, we were not able to define the robotic arm as a dynamic area of interest (AOI). Instead, the entire movement space of the arm had to be treated as a static AOI. This approach may have captured occasional fixations on the background behind the arm or may have led to arm fixations being misclassified as display fixations, when the arm moved in front of the robot’s display. Although the background contained no competing visual stimuli, likely reducing systematic misclassification, these measurement ambiguities should be considered when interpreting fine-grained eye-tracking results. Importantly, these issues were present across all experimental conditions and, therefore, are unlikely to have biased the interpretation of relative group differences.

A third limitation concerns external validity. Both studies were conducted in a controlled laboratory environment, with participants resting their heads in a chin rest to ensure stable eye-tracking data. Although the core task of predicting robot movements and interacting via a tablet resembles elements of certain industrial workflows, the experimental setting lacked many characteristics of real-world industrial environments (e.g., noise, physical workload, safety considerations, or environmental distractions). Moreover, the industrial character of the scenario was conveyed primarily through the robot and the instructions, rather than a fully immersive industrial workspace. Despite these limitations, we retained this controlled paradigm because it allowed us to isolate the effects of predictive cues on performance, trust, and visual attention with minimal confounding influences. However, this necessarily limits the generalizability of our findings. Future work should increase ecological validity by implementing more realistic scenarios, for example, by expanding the number or type of targets (e.g., tools or components), introducing collaborative dynamic interactions between human and robot, or creating settings that more closely resemble real industrial environments. Such extensions will help determine whether the performance and trust effects observed here translate to operational human-robot collaboration. Another limitation concerns the reliance on a convenience sample consisting predominantly of students, who are not representative of the typical industrial workforce. However, the primary aim of the present research was to investigate fundamental perceptual and behavioral mechanisms, such as visual attention allocation and action prediction, that are likely to be shared across user populations. To this extent, these mechanisms may generalize to industrial contexts. Nevertheless, future studies should examine whether the observed effects replicate in samples of experienced industrial workers. This concern is particularly relevant for subjective trust, as human-related characteristics such as demographics and prior experience with robots are known to influence trust^28,29^.

Another important aspect concerns our decision to include a replication study, which inevitably increases the length and complexity of this paper. Replication has gained renewed attention in psychological science due to ongoing discussions about a replication crisis^57^. Yet, despite its central role in scientific self-correction, direct replications remain exceedingly rare: recent estimates suggest that only about 0.2% of published articles between 2010 and 2021 were direct replications^58^. This highlights that replications are still undervalued, even though they carry intrinsic scientific value. In the present case, the need for replication was also substantively motivated. Prior work using similar paradigms reported improved prediction time for robotic gaze cues^16^, whereas our more recent study using the same cues failed to replicate these behavioral effects^37^. Given these inconsistent findings, the evidence for predictive gaze effects remained inconclusive. We therefore adjusted the paradigm to address earlier limitations and included a planned replication to assess the robustness of any observed effects. Including a replication therefore strengthens the methodological rigor and reliability of the reported findings.

In addition, following recommendations in the literature^51^, we conducted a mini meta-analytical aggregation across the two studies to provide an estimate of overall effect patterns. Although two studies are formally sufficient to compute a meta-analytic estimate^59^, such a small number is insufficient to draw generalizable conclusions or to establish stable effect estimates. Accordingly, the results of the mini meta-analytical approach should be interpreted with caution and not overgeneralized.

Overall, considering the present findings alongside our previous work^37^, a clearer picture emerges regarding the use of gaze cues in industrial HRI. Although humans generally exhibit an automatic tendency to follow others’ gaze^60,61^, our earlier study^37^ showed that this mechanism does not necessarily translate into immediate cue use in stressful or time-constrained interactions. Simply presenting predictive cues was insufficient for participants to notice, or benefit from them. In contrast, the current studies demonstrate that when users are explicitly informed about the meaning of predictive cues, they not only attend to them more consistently but also benefit from them in the form of faster and more efficient interaction, at least as long as the cues function correctly. This pattern is consistent with the idea that cue effects unfold across several stages: cues may first guide attention, this attentional advantage may then support explicit prediction when users treat the cues as informative, and trust may be shaped more gradually by repeated experiences of cue reliability or failure. Therefore, when errors occur, compliance with predictive cues decreases. Both studies revealed short-term declines in performance and trust following cue-action mismatches, indicating that participants temporarily disengaged from the cues and shifted their attention toward the robotic arm to verify the movement target. Importantly, both performance and trust showed signs of recovery once the cues became reliable again, highlighting the dynamic and potentially reversible nature of these effects. Together, these findings point to several practical implications. First, predictive cues, particularly gaze-like cues, can function as efficient communication signals in industrial settings by enhancing the predictability of robot actions and supporting coordination, especially in environments where verbal communication is limited (e.g., noisy factory floors). While gaze cues showed more consistent benefits over no cues, arrows seemed to facilitate prediction as well, though these effects were less reliable and less pronounced. Therefore, the practical advantage of gaze cues lies in their consistency rather than an absolute superiority over arrow cues. However, such cues must operate reliably, as cue-action mismatches may undermine both performance and trust. Second, cue mechanisms should be explicitly explained to users so that they understand their function, can effectively leverage their benefits, and are able to detect and appropriately respond to potential cue errors. Importantly, even in industrial settings where interaction primarily serves as a means to achieve a task rather than as a social goal, functionally informative, gaze-like cues can guide attention and facilitate prediction. This suggests that anthropomorphic cues retain practical value beyond purely social contexts, supporting efficient action prediction and interaction even when the social function of the cue is not central. In conclusion, this work contributes to current HRI research by suggesting that visual attention may represent one important mechanism through which predictive cues can support action prediction under uncertainty, while trust appears to depend more indirectly on the experienced reliability of those cues over time. Practically, it highlights how carefully implemented gaze cues can support efficient and predictable HRI with an industrial robot, provided their impact in cases of failures are explicitly accounted for.

## Data Availability

The datasets generated and analyzed during both studies are available in the Open Science Framework. Study I: https://osf.io/7pq6m ; Study II: https://osf.io/vtxhy/. Also, both studies were preregistered on the OSF. Study I: https://osf.io/37bwm/; Study II: https://osf.io/wd6uc.
